# Inhibition of ACSL4 ameliorates tubular ferroptotic cell death and protects against fibrotic kidney disease

**DOI:** 10.1038/s42003-023-05272-5

**Published:** 2023-09-05

**Authors:** Yue Dai, Yuting Chen, Dexiameng Mo, Rui Jin, Yi Huang, Le Zhang, Cuntai Zhang, Hongyu Gao, Qi Yan

**Affiliations:** Department of Geriatrics, Tongji Hospital, Tongji Medical College, Huazhong University of Science and Technology, Wuhan, China

**Keywords:** Cell death, Chronic kidney disease

## Abstract

Ferroptosis is a recently recognized form of regulated cell death, characterized by iron-dependent accumulation of lipid peroxidation. Ample evidence has depicted that ferroptosis plays an essential role in the cause or consequence of human diseases, including cancer, neurodegenerative disease and acute kidney injury. However, the exact role and underlying mechanism of ferroptosis in fibrotic kidney remain unknown. Acyl-CoA synthetase long-chain family member 4 (ACSL4) has been demonstrated as an essential component in ferroptosis execution by shaping lipid composition. In this study, we aim to discuss the potential role and underlying mechanism of ACSL4-mediated ferroptosis of tubular epithelial cells (TECs) during renal fibrosis. The unbiased gene expression studies showed that ACSL4 expression was tightly associated with decreased renal function and the progression of renal fibrosis. To explore the role of ACSL4 in fibrotic kidney, ACSL4 specific inhibitor rosiglitazone (ROSI) was used to disturb the high expression of ACSL4 in TECs induced by TGF-β, unilateral ureteral obstruction (UUO) and fatty acid (FA)-modeled mice in vivo, and *ACSL4* siRNA was used to knockdown *ACSL4* in TGF-β-induced HK2 cells in vitro. The results demonstrated that inhibition and knockdown of ACSL4 effectively attenuated the occurrence of ferroptosis in TECs and alleviated the interstitial fibrotic response. In addition, the expression of various profibrotic cytokines all decreased after ROSI-treated in vivo and in vitro. Further investigation showed that inhibition of ACSL4 obviously attenuates the progression of renal fibrosis by reducing the proferroptotic precursors arachidonic acid- and adrenic acid- containing phosphatidylethanolamine (AA-PE and AdA-PE). In conclusion, these results suggest ACSL4 is essential for tubular ferroptotic death during kidney fibrosis development and ACSL4 inhibition is a viable therapeutic approach to preventing fibrotic kidney diseases.

## Abstract (web summary)

Dai et al. identified that ACSL4 in kidney fibrosis increase ferroptotic precursors of tubular epithelial cells by modulating arachidonic acid (AA) and adrenic acid (AdA) of polyunsaturated fatty acids (PUFA). Inhibition of ferroptosis by targeting ACSL4 rescued tubular epithelial cells ferroptosis and alleviated kidney fibrosis, which provide more potential therapeutic approaches.

## Introduction

The incidence and prevalence of chronic kidney disease (CKD) continue to grow and are gradually emerging as one of the leading causes of mortality worldwide^1^. Kidney fibrosis is regarded as the final common pathological manifestation of chronic kidney diseases, and the process characterized by the deposition of extra cellular matrix, the proliferation of interstitial myofibroblasts, interstitial inflammatory response and loss of interstitial capillary integrity^2,3^. It is widely accepted that among the diverse cell types involved in renal fibrosis, tubular epithelial cells (TECs) act as primarily responder to pathological changes in interstitium following early injury and to produce tubulointerstitial fibrosis^4,5^. Thus, a better understanding of the mechanism of TECs in kidney fibrosis is essential for revealing therapeutic options to prevent the progression of CKD. TECs death is a hallmark feature of renal parenchymal damage, and there is increasing evidence showing that TECs death is the main mechanism of tubular damage, which is also the direct cause of kidney tubulointerstitial fibrosis after injury^6–8^. Cell cycle arrest, partial epithelial-to-mesenchymal transition (partial EMT), metabolic changes, and the loss of TECs can promote the damage in TECs^9,10^. It has been demonstrated that the depletion of TECs can be activated by regulated cell death, such as apoptosis, necrosis and pyroptosis^11–14^. With delving into the ways of cell death, ferroptosis was identified as a form of non-apoptotic cell death, which is involved in a lot of pathological processes that include kidney injury^15–19^. Several studies indicate that ferroptosis is involved in ischemia-reperfusion^20^ and folic acid^21–23^ induced kidney injury. There are only a few studies that have confirmed the core role of ferroptosis in CKD. Zhou et al.^24^ found that administrating ferroptosis inhibitor ferrostatin-1 ameliorates the extent of UUO-induced renal fibrosis in vivo Iron chelator, deferoxamine (DFO), also rescues the renal fibrosis caused by UUO by inhibiting ferroptosis^25^. Another study found that liproxstatin-1, a ferroptosis inhibitor, plays a protective role in the UUO-induced interstitial fibrosis which may respond by inhibiting the paracrine pathway of profibrotic molecules of TECs^26^. Thus, it is essential to explore the underlying molecular mechanisms of ferroptosis in TECs under renal fibrosis conditions.

Ferroptosis is driven by the iron-dependent accumulation of peroxidized lipids^15^. The peroxidation of lipids is an important step in promoting ferroptosis. Increased synthesis of polyunsaturated fatty acids (PUFA) induces lipid peroxidation during ferroptosis. Several products of lipid peroxidation including the initial lipid hydroperoxides (LOOHs) and subsequent reactive aldehydes (e.g., 4-hydroxynonenal (4-HNE)), are increased under ferroptotic stimuli conditions^27,28^. Previous studies have shown that kidney fibrosis is accompanied by the accumulation of renal lipid peroxides. Liang et al. found that 4-HNE was markedly increased in fibrotic kidney tissues after UUO challenge, suggesting that renal lipid peroxidation may be involved in the activation of ferroptosis during kidney fibrosis^29^.

Recently, some genes encoding proteins involve in lipid peroxidation for the induction of ferroptosis have been identified. Of such, acyl-CoA synthetase long-chain family member 4 (ACSL4) and lyso-phosphatidylcholine acyltransferase 3 (LPCAT3) are confirmed to participate in the biosynthesis and remodeling of PUFA- phosphatidylethanolamines (PEs) in cellular membranes^30,31^. Loss of these gene products exhausts the substrates for lipid peroxidation and raises resistance to ferroptosis. Of note, arachidonic acid (AA; C20:4) and adrenic acid (AdA; C22:4) are the prime substrates for lipid peroxidation in ferroptosis. ACSL4 first catalyzes the biochemical reaction of free AA/AdA to CoA into their acyl-CoA esters, while LPCAT3 then facilitates AA re-acylation into lysophospholipids. Doll et al.^31^ shows that the expression of ACSL4 is closely related to the sensitivity of breast cancer cells to ferroptotic death. Cancer cells with low expression level of ACSL4 are obviously resistant to ferroptotic death, and have stronger invasiveness and proliferation ability. In intestinal ischemia-reperfusion, ferroptosis of intestinal cells participated in intestinal I/R injury; inhibition of ACSL4 at the gene or pharmacological level effectively improved intestinal tissue damage induced by reperfusion by inhibiting intestinal cell ferroptosis^32^. Recent studies also found that ferroptosis-related genes, including ACSL4, was sharply increased during lung ischemia/reperfusion. Administration of ACSL4 inhibitor before ischemia can reduce the iron death degree of lung tissue and weaken the lung tissue damage caused by ischemia^33^. These studies suggest that targeted intervention of ACSL4 might be a key strategy to regulate the susceptibility of cells to ferroptotic death. However, the specific role of ACSL4 that drives ferroptosis and the downstream of the lipid signals responsible for ferroptosis activation are poorly defined in the context of fibrotic kidney diseases.

In this study, pharmacological inhibition and knockdown of ACSL4 are used to investigate its role in mediating TECs ferroptosis in kidney fibrosis. ACSL4 inhibition alleviate the kidney fibrosis by suppressing ferroptotic TECs. Then, we apply lipidomic to investigate key conditions in the process of lipid biosynthesis. The results suggest that targeting ACSL4 to inhibit AA and AdA esterification to PE is an anti-ferroptosis modality which alleviates fibrotic kidney. This may provide new targets and a theoretical basis for the treatment of CKD by targeting ACSL4 and lipid reprograming

## Results

### ACSL4 expression is upregulated during kidney fibrosis

**Fig. 1:**
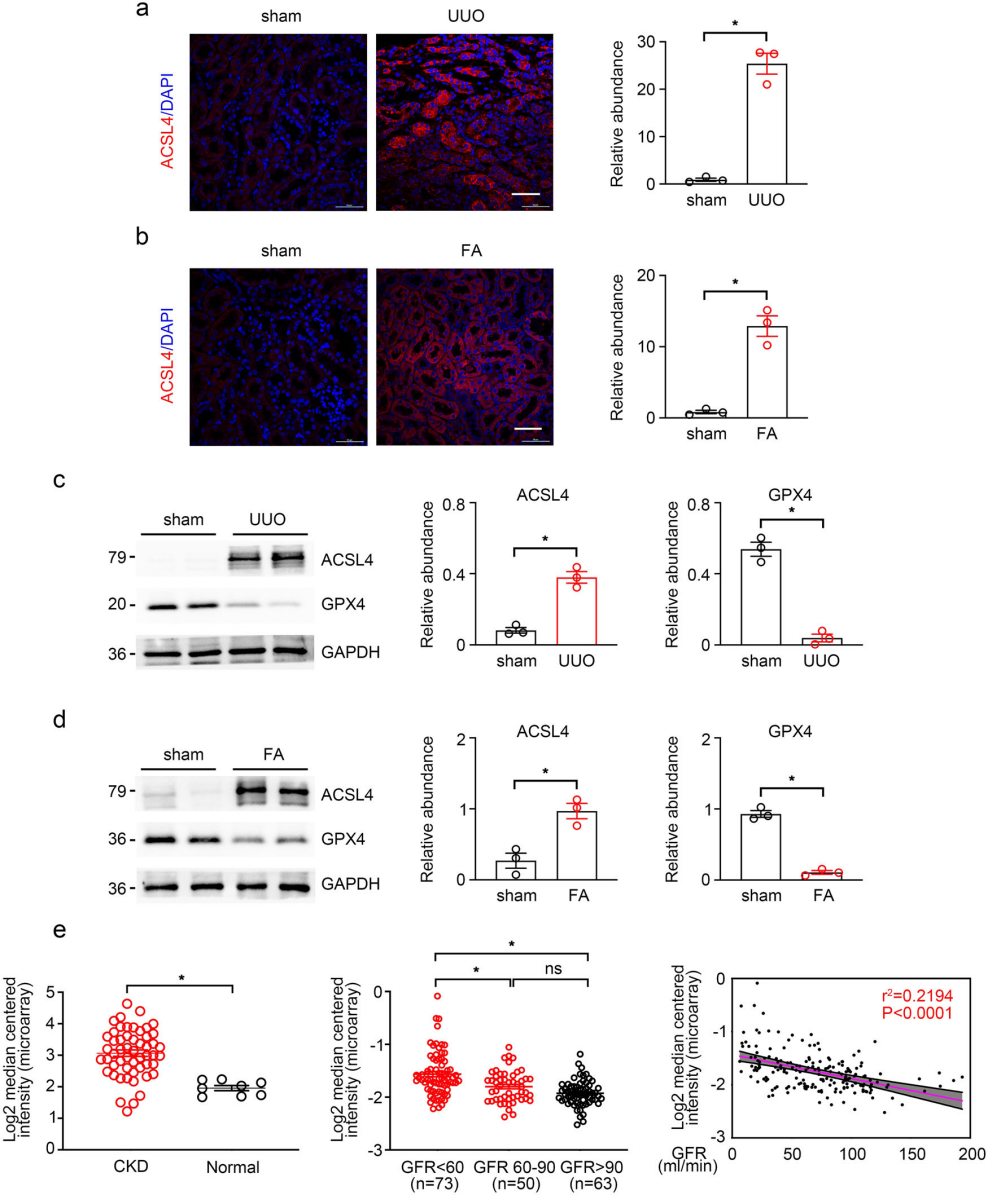
Expression of ACSL4 in fibrotic kidney. **a**, **b** The representative images (six visual fields for each sample analyzed) of immunofluorescence staining of ACSL4 in UUO and FA-induced kidney sections from indicated groups (left panel). Quantitative images to depict fluorescence intensity (Right panel). Scale bars: 50 μm. *n* = 3 mice. **c** Western-blot analysis of ACSL4 and GPX4 in UUO-treated mice separately (right panel). Schematic representation of quantitative data of indicated proteins. Representative images from three independent experiments are shown above. *n* = 3 mice. **d** Western-blot analysis of ACSL4 and GPX4 in FA-treated mice separately (right panel). Schematic representation of quantitative data of indicated proteins. Representative images from three independent experiments are shown above. *n* = 3 mice. **e** A negative correlation between tubular ACSL4 and eGFR was observed in the public Nephroseq dataset. Data are presented as mean ± SEM. *P < 0.05, ns means no statistical significance.

TECs ferroptosis can promote interstitial fibrosis^18,24^, and ACSL4 is an important regulator in the lipid metabolism of ferroptosis^34^. To determine the role of ACSL4 in fibrotic kidneys, we first examined the expression of ACSL4 in wild-type mice under the UUO and FA-treated. By the way of immunofluorescence, we found that ACSL4 was highly expressed in the renal tubules of both two renal fibrosis models (Fig. 1a, b). GPX4 is regarded as an essential regulator in ferroptosis. In our results, the protein expressions of ACSL4 and GPX4 were obviously elevated and decreased in the kidney tissues of FA and UUO-induced mice, compared with control groups as well (Fig. 1c, d). Then, Nephroseq analysis (https://www.nephroseq.org/resource /login.html) was used to explore the relationship between the mRNA expression of ACSL4 and human renal fibrosis (Fig. 1e) and found that the mRNA value of ACSL4 was shown to be obviously increased in patients with CKD. The patients were divided into three groups according to the classification of clinical manifestation, eGFR. The group of CKD (eGFR less than 60 ml/min, *n* = 73) has a tight relationship with the increased mRNA value of ACSL4 compared with the group of health control (eGFR more than 90 ml/min, *n* = 63). The results are also shown in Fig. 1e and correlated with GFR levels (r^2^ = 0.2194, *p* < 0.0001, *n* = 186). Overall, these data indicate that ACSL4 was a risk factor for kidney fibrosis.

### Inhibition of ACSL4 ameliorates kidney intestinal fibrosis by reducing ferroptosis

**Fig. 2:**
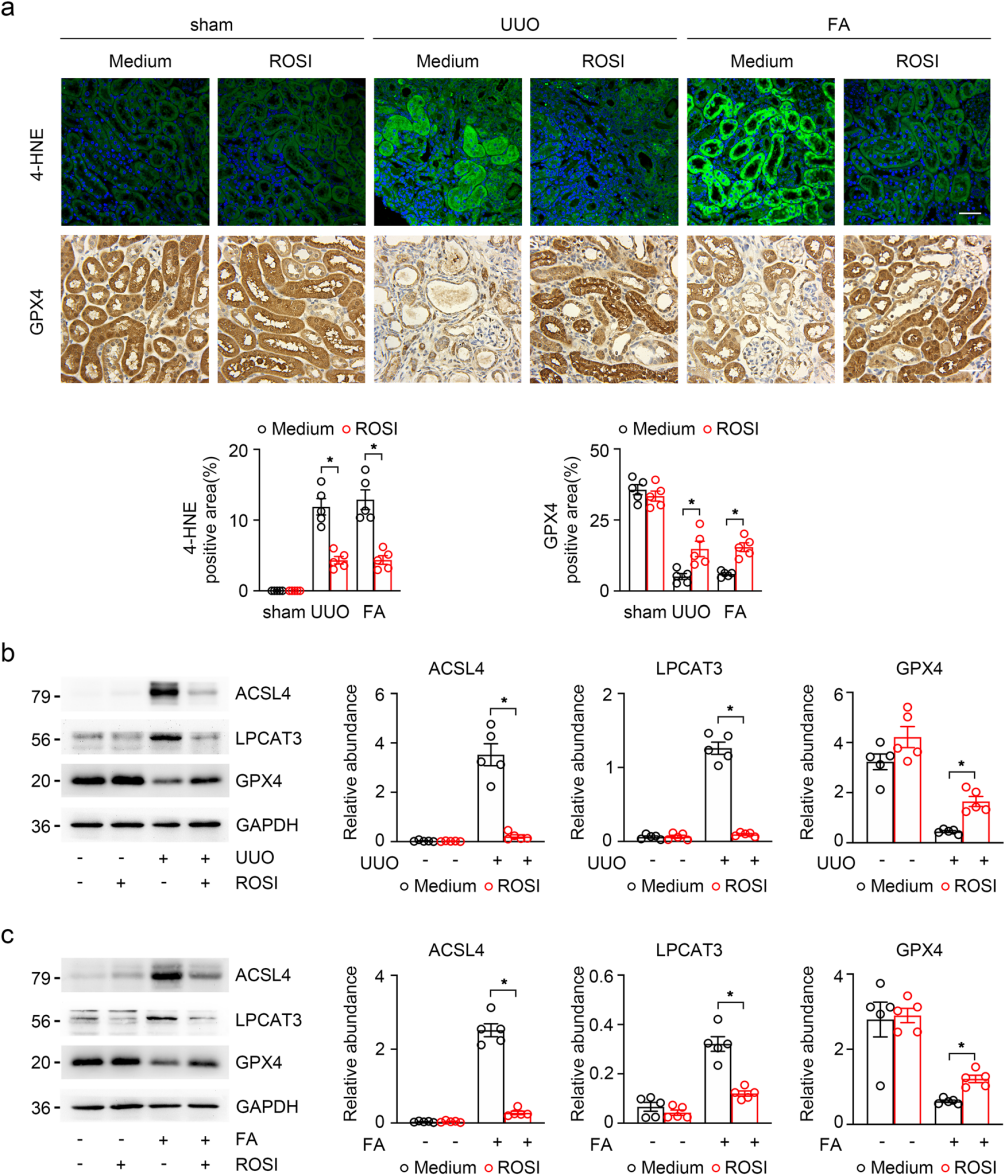
Inhibiting ACSL4 reduced TECs ferroptosis in vivo. **a** The representative image (six visual fields for each sample analyzed) of immunofluorescence staining of 4-HNE and immunohistochemistry staining of GPX4 in kidney sections under indicated conditions. Quantitative images to depict expression intensity are shown down below. Scale bars: 50 μm. *n* = 5 mice. **b** Western-blot analyzing the expression of ferroptosis-related proteins (ACSL4, LPCAT3 and GPX4) in the UUO-treated kidney samples (left panel). The relative abundances of the indicated protein expressions were normalized by GAPDH (right panel). *n* = 5 mice. **c** Western-blot analysis of ACSL4, LPCAT3 and GPX4 in FA-treated mice. Representative images from more than three independent experiments are shown in above and quantitative images are shown in the right panel. *n* = 5 mice. Data are presented as mean ± SEM. *P < 0.05, ns means no statistical significance.

To verify the role of ACSL4 in the ferroptosis of kidney fibrosis, we determined whether the inhibition of ACSL4 could abrogate ferroptosis in kidney fibrosis. As a peroxisome proliferator-activated receptor γ (PPARγ) activator, rosiglitazone (ROSI) has a specific inhibitory effect on ACSL4 independently of PPARγ signaling^35^. Our study used UUO and FA mouse models treated with ROSI by intraperitoneal injection and sacrificed on days 14 and 28 separately, to determine the role of ACSL4 inhibition in renal fibrosis. The accumulation of lipid peroxide is one hallmark of ferroptosis and 4-HNE is an α, β-unsaturated hydroxyalkenal that is produced by lipid peroxidation in cells. GPX4 can catalyze the reduction of lipid peroxidation in a complex cellular membrane environment^36^. First, in immunofluorescence, we found that ROSI reduced lipid peroxidation in UUO and FA-induced kidney fibrosis, as indicated by the decrease in GPX4 and 4-HNE on renal tubules (Fig. 2a). Subsequently, the protein level expressions of ACSL4, LPCAT3 and GPX4, core proteins in the regulation of ferroptosis, were determined in UUO and FA-induced mice treated with ACSL4 inhibitor ROSI. As shown in Fig. 2b, c, ROSI was also found to downregulate the protein levels of ACSL4 and LPCAT3, and upregulate the expression of the antioxidant GPX4.

**Fig. 3:**
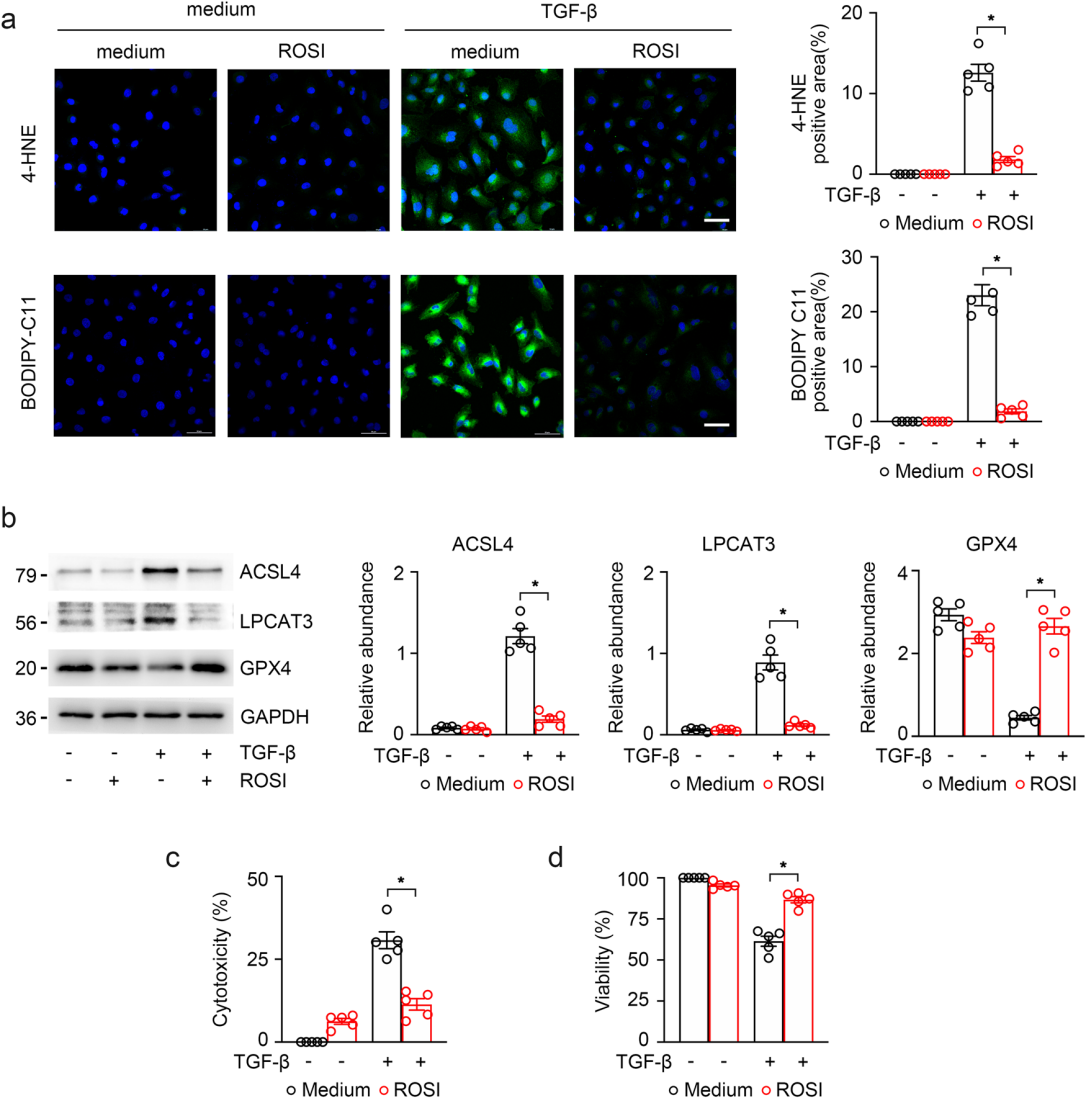
The relationship between ACSL4 inhibition and TECs ferroptosis in vitro. **a** Control and TGF-β-induced cells treated with 100 μM ROSI for 48 h were labeled with BODIPY 581/591 C11 and immunofluorescence stained with 4-HNE. Quantitative images to depict fluorescence intensity at the right panel. Scale bar = 50 μm. **b** Western-blot analysis of ACSL4, LPCAT3 and GPX4 in TGF-β-induced HK-2 cells. Representative images from three independent experiments are shown above. Schematic representation of quantitative data of indicated proteins (right panel). **c**, **d** Cell viability measured via MTT and cell cytotoxicity assayed via LDH revealed an obviously change in the death of cultured HK-2 cells in different groups. These data are calculated from more than three independent experiments. Data are presented as mean ± SEM. *P < 0.05, ns means no statistical significance.

In addition, the role of ACSL4 was also investigated in ferroptosis of TGF-β-induced fibrotic response. Measurement of reactive oxygen species (ROS) by the lipid ROS probe of C11-BODIPY 581/591 was used to measure lipid peroxidation. ROSI also reduced the high reaction of lipid peroxidation induced in renal fibrosis (fluorescence was shifted from red to green to represent reactive oxygen species) and decreased the expression of 4-HNE in TGF-β-treated cells (Fig. 3a), suggesting that ROSI inhibits lipid peroxidation under kidney fibrosis. Consistent with the in vivo results, ROSI also down-regulated the protein level expression of ACSL4 and LPCAT3, which were highly expressed in TGF-β-induced HK-2 cells, and effectually rescued GPX4 expression (Fig. 3b). We also found that HK-2 cells treated with TGF-β for 48 h induced low cell viability with MTT assay and high cell cytotoxicity with LDH released detection, which can be improved by ACSL4 inhibitor ROSI (Fig. 3c, d).

In conclusion, our results demonstrated the important role of ACSL4 in the ferroptosis of renal fibrosis and showed that inhibiting ACSL4 may reduce the occurrence of ferroptosis in renal fibrosis.

### Inhibition of ACSL4 ameliorates kidney intestinal fibrosis by reducing ferroptosis

**Fig. 4:**
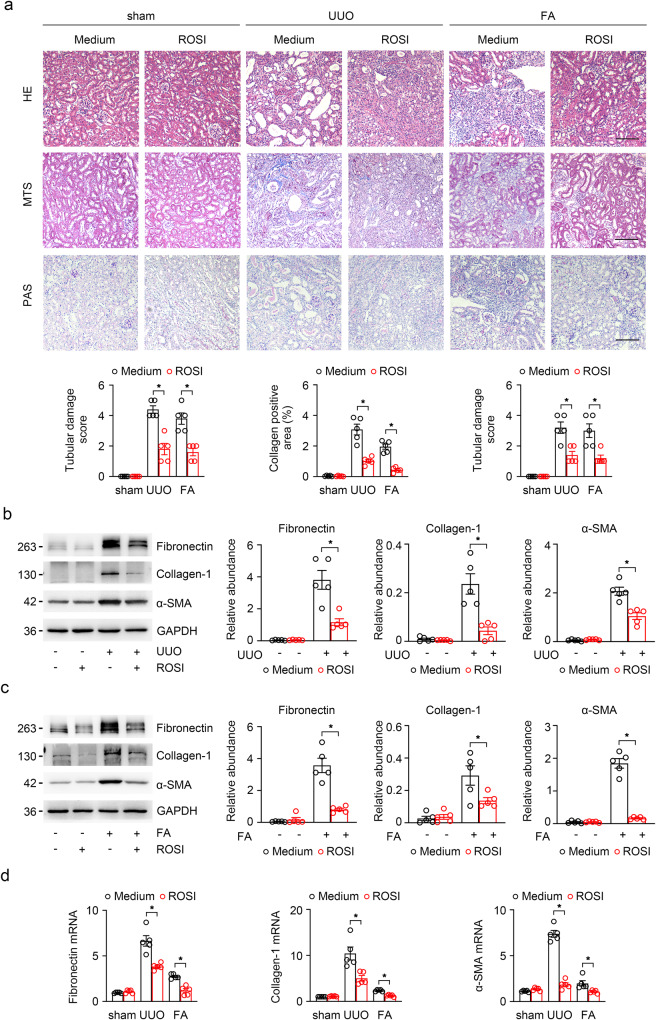
Fibrotic kidney was effectively ameliorated in UUO kidneys after inhibiting ACSL4. **a** The representative image (six visual fields for each sample analyzed) stained with HE, PAS and MTS of kidney sections from UUO, FA or sham-operated kidneys (control). Animals received ACSL4 inhibitor or vehicle as indicated. The tubular lesion and interstitial fibrosis were further presented in quantification. Scale Bar: 50 μm. *n* = 5 mice. **b** Western-blot of UUO-treated whole-kidney lysates analyzing the expression of fibronectin, collagen-I and α-SMA. Schematic representations of quantitative data of indicated proteins shown at the right panel. *n* = 5 mice. **c** Western blot analyzing the expression of indicated proteins in the FA-treated kidney samples. The relative abundance of the indicated protein expression was normalized by GAPDH (right panel). *n* = 5 mice. **d** The mRNA expression of fibronectin, collagen-I and a-SMA were analyzed by RT-qPCR in UUO and FA-treated kidneys. Representative images from three independent experiments are shown above. *n* = 5 mice. These data are calculated from more than three independent experiments. Data are presented as mean ± SEM. *P < 0.05, ns means no statistical significance.

Based on the above experimental findings, we speculated whether inhibition of ACSL4 alleviates renal fibrosis progression by decreasing ferroptosis. UUO and FA-induced kidneys exhibited severe renal structural damage such as tubulo-interstitial expansion, brush border loss and inflammatory cell infiltration (Fig. 4a). After ROSI-treated, these morphologic abnormalities were ameliorated (Fig. 4a). Additionally, in the UUO and FA mice, MTS showed substantial collagen deposition in the kidney interstitium. However, collagen fiber streaks were reduced after treated mice with ROSI (Fig. 4a). Correspondingly, the protein and mRNA expressions of fibrotic related markers, for instance, fibronectin, collagen-I and α-smooth muscle actin (α-SMA), were also evidently decreased in ROSI-treated mice with fibrotic kidney than those in UUO and FA mice (Fig. 4b–d).

**Fig. 5:**
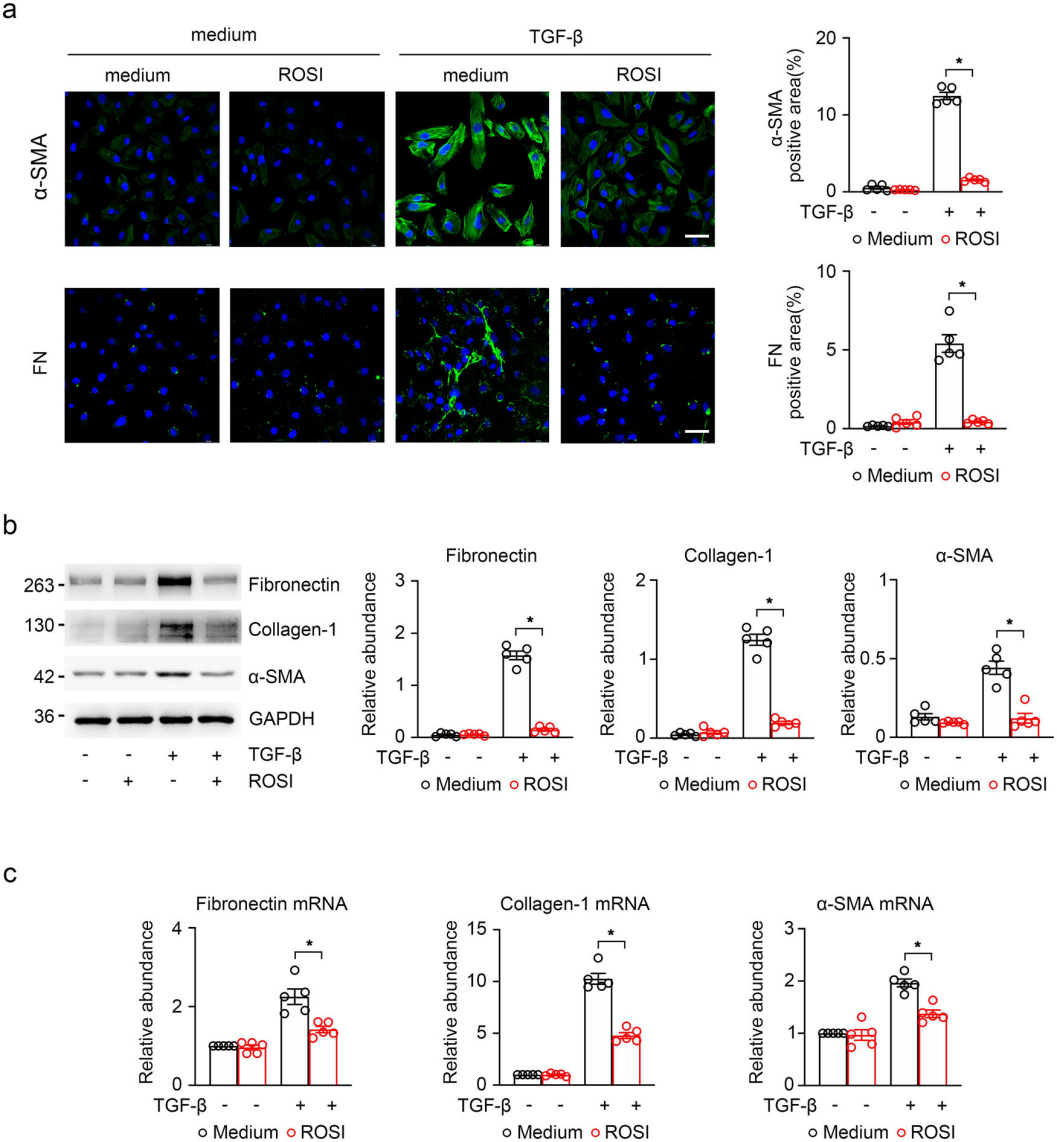
ACSL4 inhibition improved the fibrosis reaction in TGF-β-induced HK-2 cells. **a** The representative images (six visual fields for each sample analyzed) of immunofluorescence staining for fibronectin and α-SMA (green) in the indicated group. The positive areas of indicated protein were further presented in quantification. Scale bars: 50 μm. **b** Western-blot analyzing the expression of fibronectin, collagen-I and α-SMA in the TGF-β-induced HK-2 cells treated with or without ROSI (left panel). GAPDH sets as a loading control. Schematic representation of band intensity of indicated proteins (right panel). **c** RT-qPCR for fibronectin, collagen-I and α-SMA transcripts in total RNA extracts of HK-2 cells with DMSO- or ROSI-treated normal and TGF-β-treated cells. The data were calculated from three independent experiments and expressed as the mean ± SEM. Representative images from more than three independent experiments are shown above. Data are presented as mean ± SEM. *P < 0.05, ns means no statistical significance.

Moreover, we have validated the role of ROSI in the kidney fibrotic response in vitro. The results showed that fibronectin and α-SMA were considerably expressed in TGF-β-induced HK-2 cells, and remarkably expressed after ROSI treatment, which were evaluated by immunofluorescence (Fig. 5a). Likewise, we detected the expression of the fibrosis markers including fibronectin, collagen-I and α-SMA by immunoblotting and RT-qPCR, and found that the upregulated fibrosis markers were reduced after ROSI treatment in TGF-β-induced HK-2 cell (Fig. 5b, c).

In conclusion, these results support a useful effect of ACSL4 inhibition in resisting the TGF-β-induced fibrotic response.

### ACSL4 inhibition counteracts TGF-β expression and TGF-β/Smads signaling in TECs

**Fig. 6:**
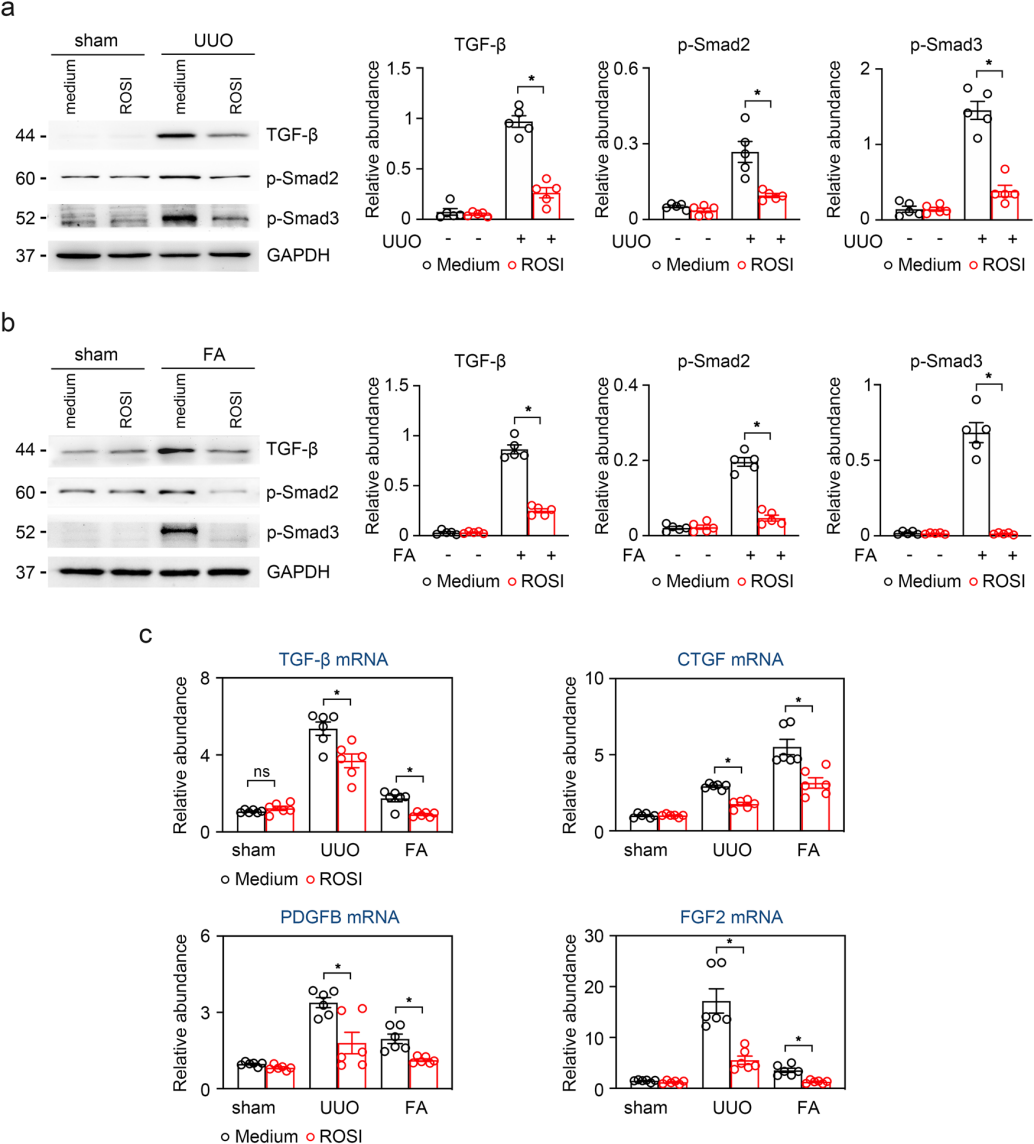
The release of profibrotic cytokines was effectively reduced in UUO and FA kidneys after inhibiting ACSL4. **a**, **b** Western-blot analyzing the expression of profibrotic cytokines TGF-β, p-Smad2 and p-Smad3 in UUO-and FA-treated kidney samples. The relative abundance of the indicated protein expression was normalized by GAPDH (right panel). *n* = 5 mice. **c** Profibrotic cytokines TGF-β, *CΤGF*, *FGF2* and *PDGFB* mRNA detected by RT-qPCR analysis in UUO and FA-treated mice. *n* = 5 mice. Representative images from more than three independent experiments are shown above. Data are presented as mean ± SEM. *P < 0.05, ns means no statistical significance.

As a major fibrosis-inducing factor, TGF-β participates in both the fibrosis signaling pathway and induces the secretion of pro-fibrotic factors, which have a profound effect on the evolution of renal fibrosis^8^. To investigate the mechanism by which ACSL4 amplifies fibrosis in the damaged kidney, we detected the expression of the TGF-β/Smads signal pathway by immunoblotting. As shown in Fig. 6a, b, inhibiting ACSL4 obviously downregulated the high expression of TGF-β, p-Smad3 as well as p-Smad2 in both UUO and FA-treated kidneys. Meanwhile, we examined the mRNA expression of some profibrotic cytokines, such as TGF-β, connective tissue growth factor (*CTGF*), fibroblast growth factor 2 (*FGF2*) and platelet-derived growth factor subunit B (*PDGFB*), and found that ROSI also attenuated the release of profibrotic cytokines in the fibrotic kidneys with UUO and FA-treated (Fig. 6c).

**Fig. 7:**
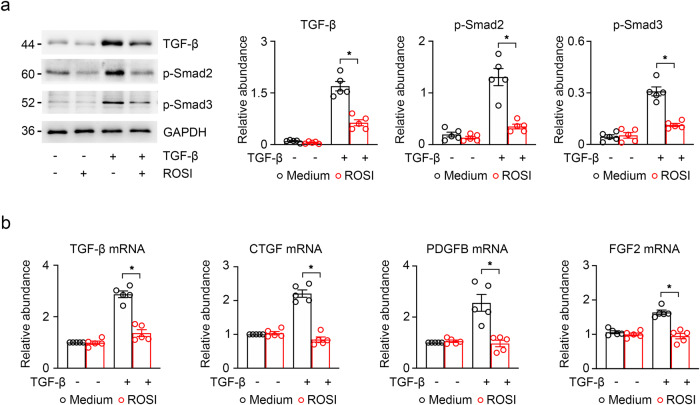
ACSL4 inhibition reduced profibrotic reaction in TGF-β-induced HK-2 cells. **a** Western-blot analyzing the expression of profibrotic cytokines TGF-β, p-Smad2 and p-Smad3 in TGF-β-induce HK-2 cells. The relative abundance of the indicated protein expression was normalized by GAPDH. Schematic representation of quantitative data of indicated proteins (right panel). **b** The expressions of profibrotic cytokines TGF-β, *CTGF*, *FGF2* and *PDGFB* mRNA were analyzed by RT-qPCR in TGF-β-induced HK-2 cells. Representative images from more than three independent experiments are shown above. Data are presented as mean ± SEM. *P < 0.05, ns means no statistical significance.

In a further step, we performed the validation in the TGF-β-induced TEC fibrosis. The results the same as in vivo showed that the protein level of the TGF-β/Smads signaling pathway (TGF-β, p-Smad3 and p-Smad2) were markedly increased in the TGF-β treated group than in the control and they were reduced in response to ROSI (Fig. 7a). Similarly, we observed that the gene level of various profibrotic cytokines (TGF-β, *CTGF*, *FGF2*, and *PDGFB*) were blunted in the TGF-β-induced HK-2 cells after ROSI treatment (Fig. 7b).

The above data suggest that ACSL4 inhibition may prevent kidney fibrosis by inhibiting TGF-β/Smads signaling pathway and reducing the release of profibrotic factors in TECs.

### Knockdown of *ACSL4* protects TGF-β-induced TECs from fibrosis by inhibiting ferroptosis and reducing profibrotic cytokines

**Fig. 8:**
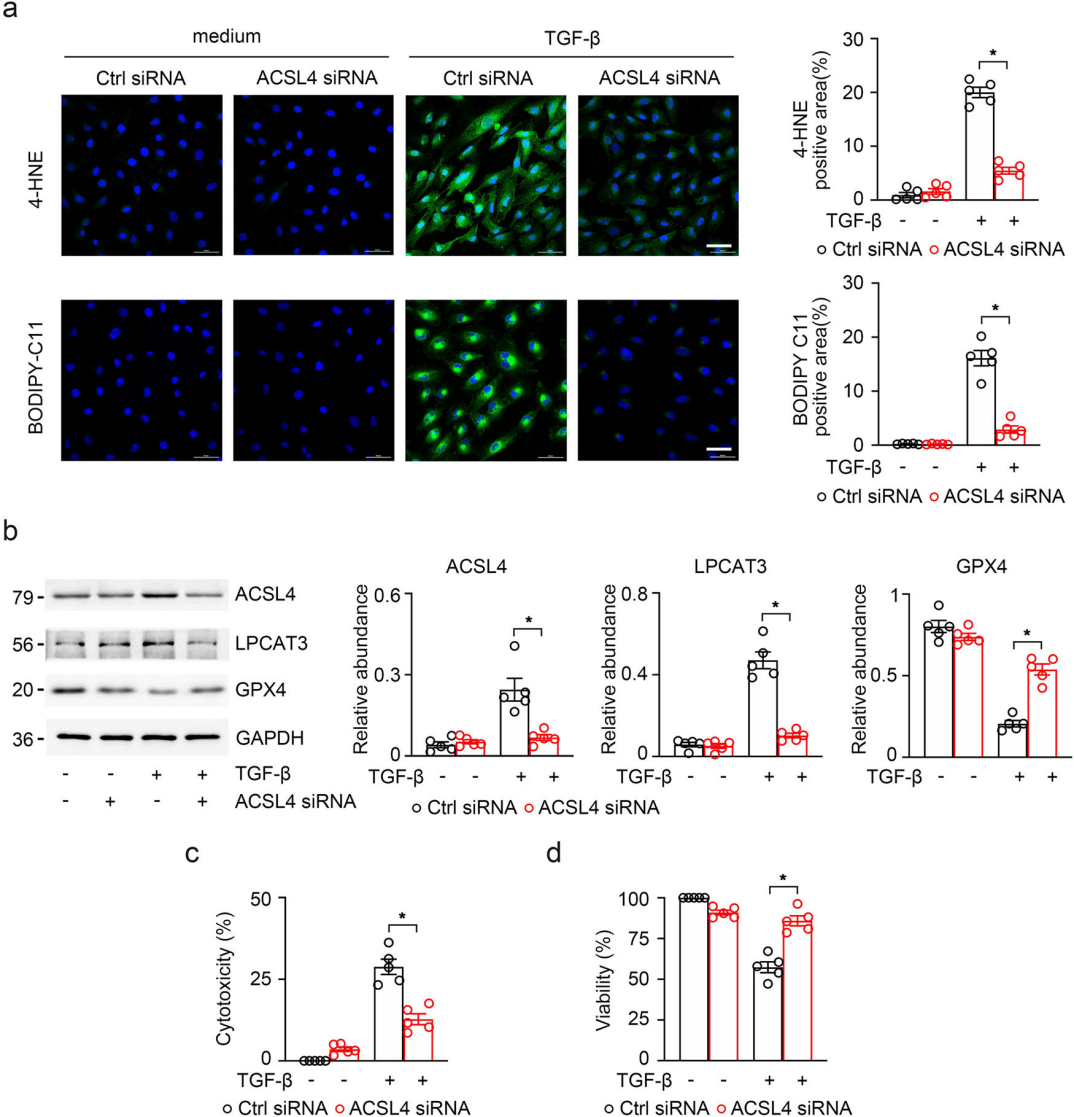
The knockdown of *ACSL4* reduced TECs ferroptosis. **a** TGF-β-induced HK-2 cells treated with ROSI or DMSO were labeled with BODIPY 581/591 C11 and immunofluorescence stained with 4-HNE. Quantitative images to depict fluorescence intensity at the right panel. Scale bar = 50 μm. **b** Western-blot analysis of ACSL4, LPCAT3 and GPX4 in TGF-β-induced HK-2 cells. Schematic representation of quantitative data of indicated proteins (right panel). Representative images from three independent experiments are shown above. *n* = 5 mice. **c**, **d** Cell viability and cell cytotoxicity were assayed by MTT and LDH respectively. Both demonstrated a effective change in TGF-β-induced HK-2 cells with the knockdown of *ACSL4*. Representative images from more than three independent experiments are shown above. Data are presented as mean ± SEM. *P < 0.05, ns means no statistical significance.

To further investigate the importance of ACSL4 in kidney fibrosis, we knocked down *ACSL4* in TECs to verify if it can rescue TGF-β-induced TECs fibrotic reaction in vitro. We transfected *ACSL4* siRNA into HK-2 treated with TGF-β and explored the role of ACSL4 knockdown in ferroptosis of kidney fibrosis. Lipid peroxidation was shown as the reduced levels of green fluorescence (BODIPY 581/591 C11) and the fluorescence intensity of 4-HNE were decreased by *ACSL4* siRNA, which were originally increased in HK-2 cells with TGF-β treatment (Fig. 8a). Then, *ACSL4*-knockdown induced a rare expression of ferroptosis regulatory proteins (ACSL4 and LPCAT3) and remarkable upregulation of GPX4 in TGF-β-treated HK-2 cells compared to those in control siRNA transfected cells as assayed by immunoblotting (Fig. 8b). The results showed that ACSL4 knockdown reduced the release of LDH and improved cell viability determined by the MTT assay (Fig. 8c, d). These results support the above conclusion that high expression of ACSL4 in renal fibrosis which induces ferroptosis in TECs.

**Fig. 9:**
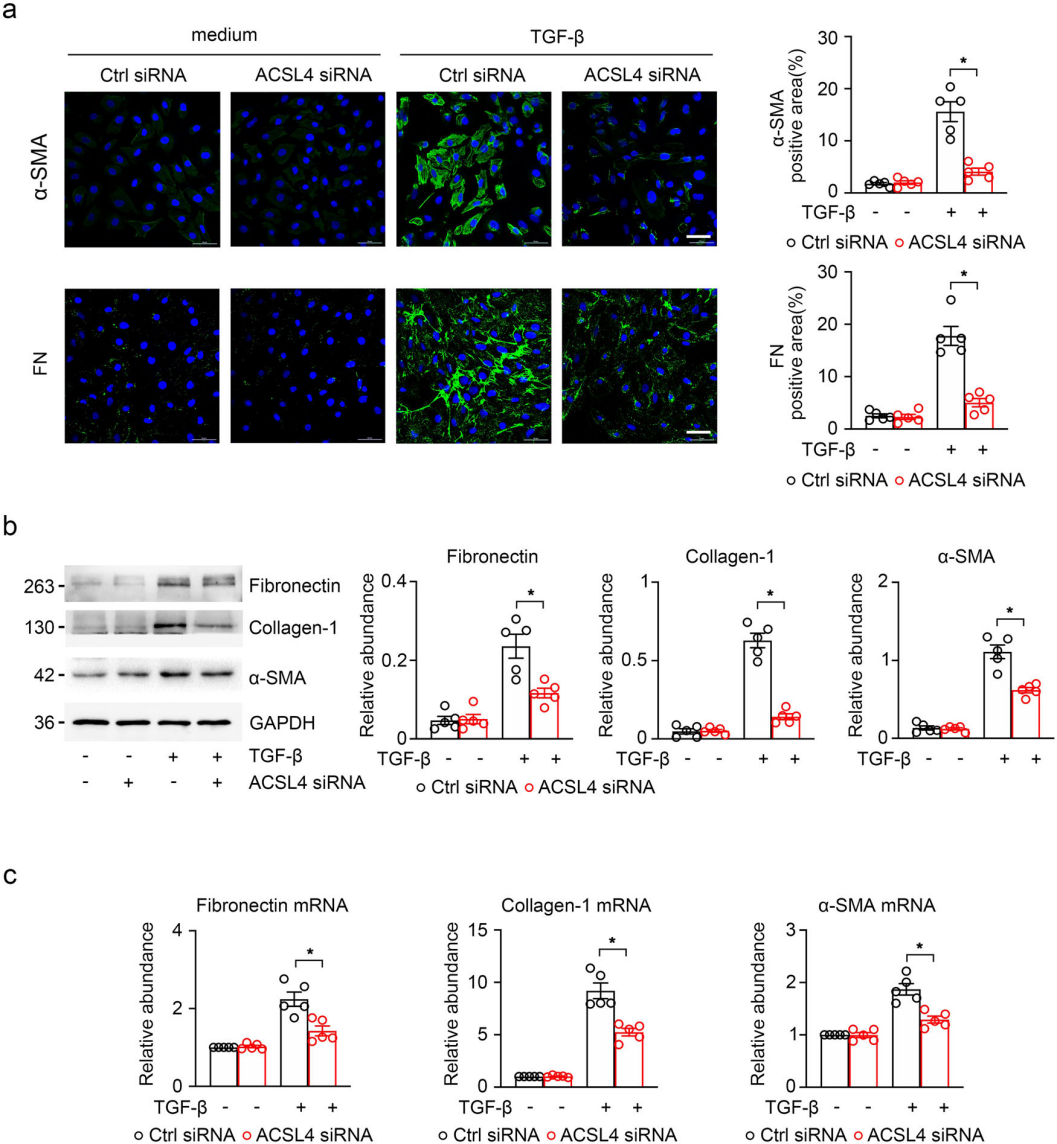
Knock *ACSL4* down alleviated the progression of kidney fibrosis. **a** The representative image (six visual fields for each sample analyzed) of immunofluorescence staining for fibronectin and a-SMA (green) in the indicated group of HK-2 cells. The positive areas of indicated protein were further presented in quantification (right panel). **b** Western-blot analyzing the expression of fibronectin, collagen-I and α-SMA in the TGFβ-induced HK-2 cells with or without the knockdown of *ACSL4* (right panel). GAPDH sets as the loading control. Schematic representation of band intensity of indicated proteins (left panel). **c** The expression of fibronectin, collagen-I and α-SMA detected by RT-qPCR in TGF-β-induced HK-2 cells with or without the knockdown of *ACSL4*. Representative images from more than three independent experiments are shown above. Data are presented as mean ± SEM. *P < 0.05, ns means no statistical significance.

Subsequently, we explored whether knockdown of *ACSL4* could attenuate renal fibrosis via blocking ferroptosis. The results showed that the fibrotic alterations in HK-2 cells under different treatments were detected by immunofluorescence and striking decreases in fibrosis makers (fibronectin and α-SMA) were found in TGF-β-induced HK-2 cells after ACSL4 knockdown (Fig. 9a). As shown in Fig. 9b, the results indicated that the knockdown of *ACSL4* suppressed the expression of the fibrotic makers (fibronectin, collagen-I and α-SMA) in HK-2 cells with TGF-β treatment. Following, we examined the mRNA level of fibronectin, collagen-I and α-SMA by RT-qPCR and found that fibrotic makers in TGF-β-induced HK-2 cells showed a greatly high level. It can be concluded that *ACSL4* siRNA transfection protected HK-2 cells against the progression of fibrotic (Fig. 9c).

The above data suggest that *ACSL4* knockdown may delay the progression of renal fibrosis by aborting ferroptosis.

### ACSL4 generates ferroptotic precursors by regulate esterification of AA-PE and AdA-PE

It has been shown that ACSL4 regulates ferroptosis by specifically esterifying AA and AdA to phospholipids of cell membranes for lipid biosynthesis^31^. Moreover, one study verified that AA-PE and AdA-PE, but not others, were identified as precursors of ferroptotic signals^28^. As mentioned before, we had demonstrated that ACSL4 may aggravate kidney fibrosis by promoting ferroptosis and explored how ACSL4 alters the sensitivity to ferroptosis in renal fibrosis by changing the lipid composition of phospholipids of the cell membrane.

**Fig. 10:**
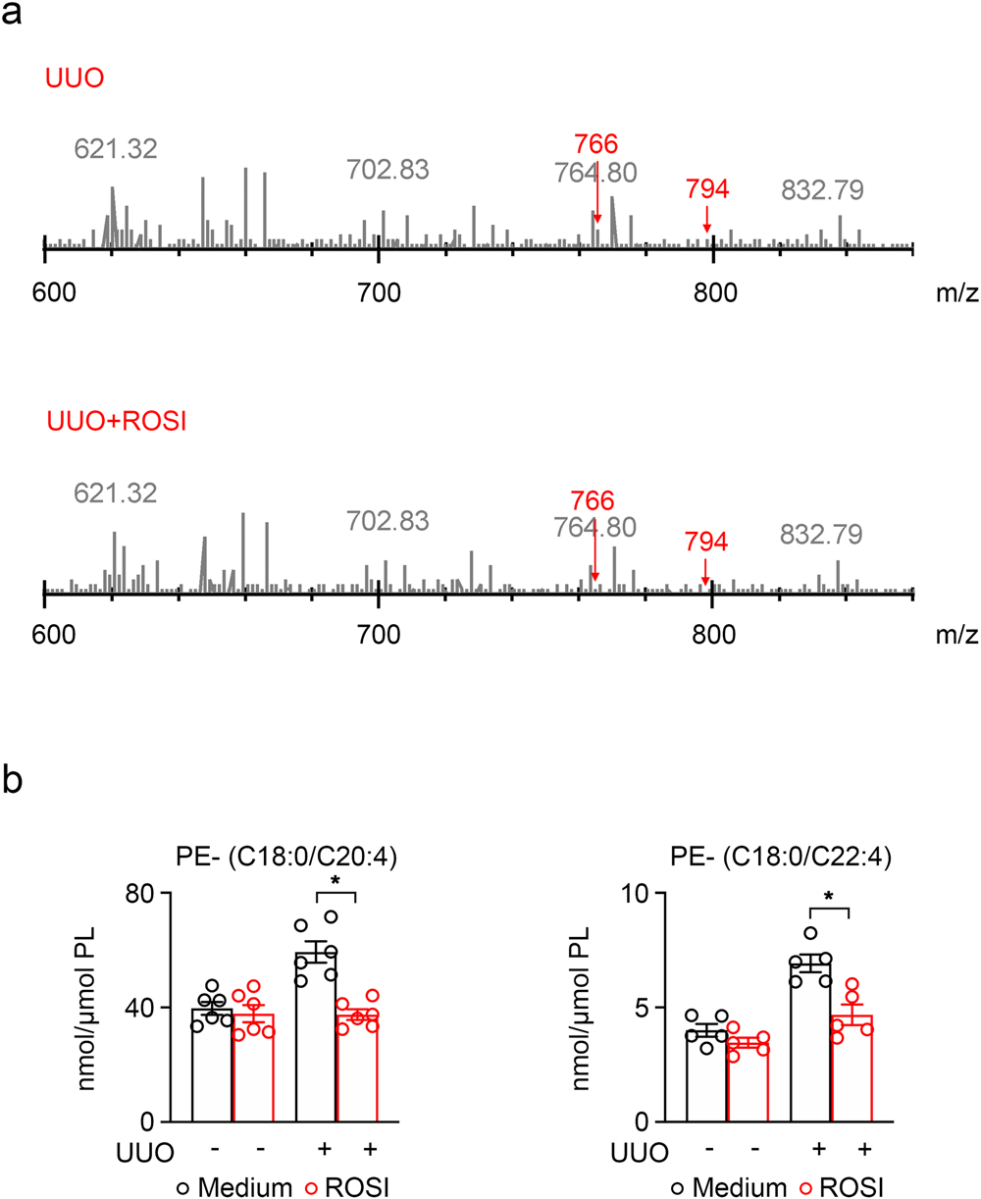
ACSL4 inhibition decreased the PE ferroptotic precursors in kidney fibrosis response. **a** Quantitative assessment of PE molecular species (C18:0/C20:4 and C18:0/C22:4) in UUO kidney treated with ROSI. Data are presented as mean ± SEM. *n* = 6 mice. **b** Levels of AA-PE (*n* = 6 mice) and AdA-PE (*n* = 5 mice) in UUO kidney with or without ROSI. Data are presented as mean ± SEM. *P < 0.05, ns means no statistical significance.

Therefore, we want to explore the details of ferroptotic precursors phospholipids during this process. We performed lipidomic LC-MS/MS analysis of kidney tissues from UUO mice and ROSI-treated UUO mice. Overall, we detected 102 different types of PE in the fibrotic kidney of mice.We observed that C18:0/ C20:4 and C18:0/C22:4 (AA-PE and AdA-PE) were markedly upregulated in UUO mice compared to WT mice and the high level of AA-PE and AdA-PE in UUO mice were markedly reduced after rosiglitazone treatment (Fig. 10a, b).

Overall, the above results indicate that ACSL4 inhibition in kidney fibrosis is accompanied by a reduction of ferroptotic precursors AA-PE and AdA-PE which are utilized as substrates in lipid peroxidation to reduce the ferroptosis cascade reaction.

## Discussion

Accumulating studies have found that ferroptosis contributes to numerous diseases, such as AKI and CKD^16,25,37^. However, the precise mechanisms of ferroptosis mediate fibrotic kidney still require further elucidation, our study emphasizes the importance of ACSL4 in this process. Findings from our study revealed that ACSL4, as the lynchpin in the reprogramming of lipid metabolism, aggravates renal fibrosis by activating endogenous ferroptotic mechanisms. In previous studies, it was found that renal tubular epithelial cell death, such as necroptosis and pyroptosis, participated in the pathogenesis of renal fibrosis. In this study, we found that the fibrotic kidney diseases, both in UUO/FA-induced mouse model and the TGF-β-induced HK-2 cell model, were associated with increased ferroptosis and upregulation of ACSL4. From this, we inferred that ferroptosis of TECs may be one of the potential mechanisms of renal fibrosis, and inhibition of ferroptosis by targeting ACSL4 may be an effective treatment for alleviating renal fibrosis.

ACSL4 is the enzyme of the long-chain acyl coenzyme A synthase family, which regulates lipid biosynthesis by activating PUFAs^38^. As the main substrates of lipids peroxidation in ferroptosis, PUFAs are susceptible to undergoing oxidation and produced hydroperoxides of PUFAs (PUFA-OOH)^39^. It is necessary for the execution of ferroptosis and which can be enhanced by ACSL4. ACSL4 also promotes PE of membrane production by esterifying PUFAs to PUFA-CoA, thereby participating in the occurrence of lipid metabolism reprogramming in ferroptosis^31^ ACSL4 is the crucial regulator to mediate ferroptosis through the reprogramming of lipid metabolism^31,40^ and participates in the pathological process of various diseases under intensity, heart, lung and muscel^28,32,33,41–43^. Recent studies have found that ACSL4-mediated ferroptosis is also implicated in kidney disease^44,45^. Wang et al.^45^ demonstrated that ACSL4 plays a protector in the ferroptosis of AKI by knocking out ACSL4 in TECs of mice In human acute tubular injury, the expression of ACSL4 in TECs is upregulated, accomplished with the elevation of serum creatinine and blood urea nitrogen and the declined level of eGFR^46^. Muller et al.^47^ and Yuan et al.^34^ suggested that the expression of ACSL4 can be served as a biomarker to help predict the relationship between ferroptosis and the pathological mechanism of disease. The findings of Zhou et al.^24^ and Wang et al.^25^ indicated that the execution of ferroptosis can aggravate the progression of CKD. The kidney is also regarded as an important site that actively participates in the systemic regulation of lipid metabolism. Thus, we found that ACSL4 is upregulated in the kidney tissue of CKD through the analysis of the Nephroseq-database and the experiments of renal fibrosis in vivo and vitro, and is also involved in the ferroptosis-related fibrotic kidney. These results indicate that ACSL4 is a crucial inducer in the ferroptosis of renal fibrosis.

Cell death and partial EMT caused by injured TECs are the key triggers in renal fibrosis^9,18,24,48–51^. The studies of nucleus RNA sequencing have identified specific maladaptive/profibrotic proximal tubule cells undergoing chronic injury^49–51^. Such maladaptive/profibrotic proximal tubule states can contribute to the critical processes of interstitial fibrosis and the progression of CKD through the induction of dedifferentiation, G2/M arrest, senescence, partial EMT and pro-fibrotic cytokine secretion^9,51,52^. The partial EMT program is in an epithelial-mesenchymal intermediate state, which leads to the dedifferentiation of renal epithelial cells and promotes kidney fibrosis but preserves the integrity of the renal tubules^52,53^. TGF-β is one of the most critical profibrotic cytokines which stimulates the secretion of profibrogenic cytokines, such as *CTGF*, *FGF2* and *PDGFB*. TGF-β/Smads signaling was demonstrated as the activation that induced partial EMT phenotypes in TECs^9,54^. To explore whether ACSL4 aggravated renal fibrosis by the TGF-β/Smads signaling and the variety of profibrotic factors, ACSL4 inhibitor ROSI was treated in TGF-β-induced TECs and mouse models. We found that inhibition of ACSL4 by ROSI alleviated the fibrotic TECs and reduced the creation of profibrotic factors by abolishing ferroptosis. These results indicate that ACSL4 is a crucial factor that accelerates the progression of renal fibrosis through activating TGF-β/Smad signal pathway and releasing several profibrotic factors.

Thiazolidinediones, such as rosiglitazone (ROSI) and pioglitazone (PIO), are specific PPARγ full agonists that are used as insulin sensitizers and are widely used clinically for the treatment of diabetes^55^, but their clinical application is restrictively prescribed by giving their increased risk of cardiovascular disease and fracture, and was prohibited in patients with severe CKD. Because ROSI powerfully and specifically inhibits ACSL4 but no other ACSL isoforms, ROSI has been extensively used as a ferroptosis inhibitor in ferroptotic-related studies^56^. Our in vitro results validate that the pharmacological inhibition of ACSL4 by ROSI is as same effective as *ACSL4* knockdown in ameliorating tubular ferroptosis and antagonizing renal fibrosis, but it cannot be excluded that ROSI may be involved in regulating kidney fibrosis by modulating PPARγ activity^57^. Recently, several repurposing drugs have been identified that specifically inhibit ACSL4, such as Abemaciclib and PRGL493, but their potential role and/or side effects in ferroptotic-related diseases, especially in acute or chronic kidney diseases, remain to be further investigated^58,59^.

Excessive PUFAs are prone to induce lipid peroxidation and also are a risk factor in patients with CKD^60,61^. GPX4 is the crucial antioxidant that controls ferroptosis and alleviates lipid hydroperoxides within biological membranes, and its decrease can be caused by the dysregulation of the redox system caused by the high expression of ACSL4^35^. We validated this solution at the pharmacological and genetic levels and found that inhibition of ACSL4 can reduce lipid peroxidation, thereby elevating the expression of GPX4. AA and AdA in PUFAs are more sensitive to lipid peroxidation, which can be preferentially acylated by ACSL4 and then participate in the synthesis of phosphatidylethanolamine under the action of LPCAT3, and the product becomes the important ferroptotic signals^40,62^. Doll et al. analyzed the Redox phospholipidomics in GPX4-deficient mouse embryonic fibroblasts by LC-MS/MS identification and found that PE molecular species with C18:0 fatty acids and C20:4 (AA) or C22:4 (AdA) fatty acids were decreased in *ACSL4* KO cells^31^. By lipidomic analysis, our study also showed that the expressions of AA-PE (C18:0/C20:4) and AdA-PE (C18:0/C22:4) were decreased after ROSI treatment in UUO. Therefore, we suggest that renal fibrosis may be caused by ACSL4-induced incrassation of AA-PE and AdA-PE in membrane phospholipids which are the ferroptotic precursors. The results demonstrated that ACSL4 is involved in the esterified oxidated PUFAs in fibrotic kidney and highlighted the importance of inhibiting ACSL4 as a blocker of ferroptotic lipid signaling which provides new pharmacological targets.

In conclusion, our present findings demonstrate that ACSL4 activation during kidney fibrosis development contributes to tubular ferroptosis, and inhibition of ferroptosis by targeting ACSL4 rescue chronic kidney injury. Identifying the PUFAs especially AA and AdA, which are modulated by ACSL4, may provide new insights into understanding the signaling pathway involved in ferroptosis of TECs during kidney fibrosis. The protective effects to treat ferroptotic TECs in CKD deserve further exploration and will develop more novel therapeutic approaches for CKD.

## Methods

### Animals

This study have complied with all relevant ethical regulations for animal testing. All handlings and experimental procedures of mice were performed in accordance with the guidelines of the National Health and Medical Research Council of China, and the protocol was approved by the animal ethics review board of Tongji Medical College in Huazhong University of Science and Technology (Permit Number: S 2670).

Male C57BL/6J mice were fed in a specific pathogen-free (SPF) environment in the laboratory animal center of Tongji Medical College. Animals were given free access to normal diet and acclimatized in a ventilated temperature-controlled room (24 °C), with a regular 12 h light/dark cycle. Male 8–12-week C57BL/6 mice were used in the follow experiments. For unilateral ureteral obstruction (UUO) model, mice underwent left UUO surgery, the left mid-ureter of mice was exposed via a lateral incision and obstructed it twice with 4–0 silk sutures.

For folic acid administration model, mice were intraperitoneally injected with a single dose of folic acid (250 mg/kg body weight dissolved in 300 mM NaHCO3). The mice were intraperitoneally treated with ROSI (0.5 mg/kg/day) at 1 h prior to handlings of UUO and FA, and continuously injected daily for UUO and FA duration. After 14 days for UUO model and 28 days for FA model, kidney tissues were collected for the further experiments.

### Cell lines and culture conditions

HK-2 (human kidney tubular cell) was obtained from China Centre for Type Culture Collection (CCTCC, China), and maintained in DMEM-F12 (Biological Industries, USA) medium containing 10% fetal bovine serum (FBS) and 1% penicillin-streptomycin. Cells were incubated in a humidified atmosphere of 95% air and 5% CO2 at 37 °C. At the case of cells treatment, recombinant human TGF-β (Peprotech, Rocky Hill, NJ) was added at cells for 48 h, with the concentration of 20 ng/ml. 100 μM rosiglitazone (Sigma-Aldrich, USA) treated cells for 48 h.

*ACSL4* siRNA and control siRNA was purchased from Qiagen (Germany). Transfection was performed using HiPerFect transfection (Qiagen, Germany) according to the manufacturer’s protocol. The efficiency of transfection was assessed by the protein expression. The sequence used for knockdown of *ACSL4* in this study was: 5′-TTGGAGCGATTTGAAATTCCA -3′. HK-2 cells treated with or without TGF-β, as well as the cells transfected with *ACSL4* siRNA for 48 h.

### Cell viability and cytotoxicity assay

HK-2 cells were cultured with the optimal cell concentration of 1 × 104 cells/well in a 96-well plate. During TGF-β induced fibrosis, HK-2 cells were treated with rosiglitazone in different groups for 48 h. For the *ACSL4* siRNA treatment experiments, HK-2 cells were treated with control siRNA, *ACSL4* siRNA or TGF-β for 48 h after plating. Cell viability was measured by MTT (KeyGEN, KGA311, China). MTT were diluted by buffer and added at 96-well plate with 50 μL/well to incubate 4 h at incubators. Absorbance was detected at 490 nm after treatment with 150 μL of DMOS reagent for 1 h. Cell cytotoxicity was detected by LDH (Roche, 04744926001, USA). To determine the LDH activity, we conducted cells according to the manufacturer’s instructions. The absorbance was read at 490 nm and caculated to get the percentage cytotocicity by the following equation: cytotoxicity(%) = (value of samples − low control)/(high control − low control) × 100%.

### Cell immunofluorescence assay

HK-2 cells were cultured in 6-well plate format for cell immunofluorescence analysis. After treatment of rosiglitazone (100 μM) and TGF-β (20 ng/ml) for 48 h, the medium was removed and cells were cleaned three times with PBS. Cells in coverslips of 6-well plate were fixed in 4% paraformaldehyde for 15 min, and then blocked with 10% goat serum (Boster, AR0009, China) for 1 h before incubation with primary antibodies against 4-HNE (1:100 dilution), Fibronectin (1:250 dilution) and α-SMA (1:250 dilution) at 4 °C for 24 h. After incubation, coverslips were rinsed three times with PBS for 5 min and incubated with the secondary antibodies Dylight 488 goat anti-rabbit IgG (Abbkine, California, USA) and Dylight 594 goat anti-mouse IgG (Abbkine, California, USA) for 60 min at room temperature. After antifade mounting medium with DAPI (Beyotime, P0131, China), samples were observed under confocal microscopy (Nikon C2, Japan) and fluorescence intensity was analyzed using ImageJ software.

### BODIPY 581/591 C11 analysis

HK-2 cells were treated with rosiglitazone (100 μM) and TGF-β (20 ng/ml) for 48 h at 6-well dishes containing 25 mm coverslip per well. After treatment, the medium were removed and cells were washed three times with sterile PBS. Complete medium was used to prepare BODIPY 581/591 C11 (Thermo Fisher Scientific, D3681, USA) working solution with the concentration of 5 μM. 6-well dishes were added 1 ml per well and incubated at 37 °C for 30 min in the dark. At the end of the treatment, we removed the medium and cleaned cells three times with PBS. Glass coverslips with cells were fixed in 4% paraformaldehyde for 15 min. Subsequently, coverslips were transferred to glass microscope slide with the antifade mounting medium with DAPI (Beyotime, P0131, China). The images were captured using confocal microscopy (Nikon C2, Japan). To quantify the intensity of oxidization, the background was corrected by subtracting the red or green fluorescence in cell-free areas and then calculated the ratio of the green fluorescence to red and green fluorescence.

### Histological analysis

Excised kidney tissues were fixed in 4% paraformaldehyde, embedded in paraffin and sectioned for the following experiments. Sections were stained with hematoxylin-eosin (HE), periodic acid-Schiff (PAS), and Masson’s trichrome staining (MTS) according to the standard protocols. We evaluated the degree of tubular atrophy in line with the renal tubular damage score, which were based on cast formation, tubular dilation, brush border loss and interstitial fibrosis, with scores corresponding to the following percentages of renal tubular damage: 0, 0%; 1, ≤10%, 2, 11 to 25%; 3, 26 to 45%; 4, 46 to 75%; and 5, ≥76%. In each sectiones, damaged areas were determined in the successive field of entire cortical and juxtamedullary areas. The total damaged areas under examination using Image J software (National Institutes of Health, Bethesda, MD) assisted image analysis. MTS was analyzed semi-quantitatively by quantifying the blue-stained areas of 6 random different views.

### Immunohistochemistry (IHC) and immunofluorescence (IF) assay

We performed immunohistochemistry and immunofluorescence in paraffin sections using the steam-based antigen retrieval technique. Deparaffined sections were used for IHC of GPX4 (Abcam, ab125066, 1:100 dilution, Britain) and IF of 4-HNE (Abcam, ab46545, 1:100 dilution, Britain), α-SMA (Abcam, ab124964,1:250 dilution, Britain) and Fibronectin (Abcam, ab45688, 1:250 dilution, Britain). Images of IF were acquired on confocal microscopy (Nikon C2, Japan). The samples of IHC were examined using the microscope (Mshot, China). The intensities of the expression of the target gene were detected by Image J software (National Institutes of Health, Bethesda, MD).

### Real-time PCR analysis

RNA was extracted from harvested HK-2 cells and kidney tissues using FastPure Cell/Tissue Total RNA Isolation Kit (Vazyme, RC11201, Chinan) and reversely transcribed into cDNA by ReverTra Ace qPCR RT kit (Toyobo, FSQ-101, Japan). The system of real-time polymerase chain reaction (RT-PCR) was mixed by SYBR Green Master and gene-specific primers, and RT-PCR was run in the ABI Step One Plus system (Applied Biosystems, USA). The mRNA expression levels of target genes were normalized and analyzed using the ΔΔCt method. The primers used in this study are listed in Supplementary Information (Supplementary Table 1).

### Western blot and antibody

Cells and kidney tissues were lysed by sonication in RIPA buffer containing 1% PMSF and 1% protease inhibitor. The protein extracts were prepared after measuring the protein concentration by BCA (Boster, AR0146, China) method. Protein samples were measured by western blotting of electrophoresis in 10% SDS-PAGE gel and transferred with PVDF membrane (Millipore, USA). Skim milk was used to block nonspecific signals at room temperature for 1 h. The membranes were incubated by primary antibodies against GPX4 (Abcam, ab125066, 1:3000 dilution, Britain), ACSL4 (Santa Cruz, SC393906, 1:1000 dilution, USA), LPCAT3(Abcam, ab232958, 1:500 dilution, Britain), Fibronectin (Abcam, ab45688, 1:5000 dilution, Britain), α-SMA (Abcam, ab124964,1:2000 dilution, Britain), Collagen-I (Proteintech, 14695-1-AP,1:1000 dilution, USA), TGF-β1 (Abcam, ab215715, 1:1000 dilution, Britain), p-Smad2 (CST, 3104S,1:1000 dilution, USA), p-Smad3(CST, 9520TS,1:1000 dilution, USA) and GAPDH (Promoter, 1:25000, China). After washing, membranes were incubated with Horseradish peroxidase-conjugated anti-mouse and anti-rabbit (Promoter, 1:5000 dilution, China) at room temperature for 1 h. Immunoblots were imaged on the UVP imager (UVP, USA). Gray values of specific bands were quantified by ImageJ software.

### Separation and detection of Lipidomic

A LC-MS system consisting of Waters 2D UPLC (Waters, USA) and Q Exactive high resolution mass spectrometer (Thermo Fisher Scientific, USA) was used for lipids separation and detection. 800 μL of extract (dichloromethane/methanol = 3/1, v/v) and 10 μL of SPLASH internal standards solution were added in the 1.5 mL Eppendorf tube contained the sample to grind. Supernatant of the sample was taken for lyophilization and econstituted with 200 μL of reconstitution fluid (Isopropanol/Acetonitrile/Water = 2/1/1, v/v/v). After ultrasonic treatment and shaking, the supernatant was placed in a 1.5 mL vial. To evaluate the repeatability and stability of LC-MS/MS analysis, 20 μL of supernatant of each sample was mixed into a QC sample. Lipidomic were analyzed by phase LC-MS/MS. Data of positive-ion and negative-ion mode was acquisited respectively to improve the lipid coverage.

### Analysis of lipidomics by LC-MS/MS

A CSH C18 column (1.7 μm 2.1*100 mm, Waters, USA) was used in this study. Under positive ion mode, the mobile phase consisted of solvent A (60% acetonitrile aqueous solution + 0.1% formic acid + 10 mM ammonium formate) and solvent B (10% acetonitrile aqueous solution + 90% Isopropanol + 0.1% formic acid + 10 mM ammonium formate). Under negative ion mode, the mobile phase contained solvent A (60% acetonitrile aqueous solution + 10 mM ammonium formate) and solvent B (10% acetonitrile aqueous solution + 90% Isopropanol + 10 mM ammonium formate). The column was eluted in the gradient elution conditions. The flow rate was 0.35 mL/min. The column oven was maintained at 55 °C. The injection volume was 5 μL. A Q Exactive mass spectrometer (Thermo Fisher Scientific, USA) was used to obtain MS1 and MS2 data. The MS scan method was in the range of m/z 200–2000, The MS1 resolution was 70,000 and the maximum injection time was 100 ms. According to the precursor ion intensity, Top 3 ions were selected for MS2 analysis, MS2 resolution was 17,500, maximum injection time was 50 ms, and collision energy were set as: 15, 30 and 45 eV. The parameters of ESI were sheath gas of 40 L/min, aux gas of 10 L/min, spray voltage of 3.80 in positive ion mode and of 3.20 in negative ion mode, capillary temperature of 320 °C and aux gas heater temperature of 350 °C.

### Pretreatment of lipidomics data

Lipidomics data processing by LipidSearch 4.1 that first conducted identification and peak extraction for each sample, and then peak alignment for all samples. The BGI’s own metabolomics software package metaX^63^ was used for statistical analysis. PCA model was used to observe the inter-group differences of the samples. The variable importance in projection values of the first two principal components of the PLS-DA^64,65^ were used to screen the differential lipid molecules by combining with fold change and student’s t test.

### Statistics and reproducibility

All data in this study were performed more than or equal to 3 independent replicate experiments, and expressed as mean ± SD. Statistical analysis was conducted using the GraphPad Prism 7.0 (GraphPad Software Inc. San Diego, CA) and SPSS 20.0 software. To establish statistical significance, Student’s *t* test was used for two-group comparisons and two-way ANOVA followed by Bonferroni’s post hoc test used to compare more than two groups. *P* values < 0.05 were considered statistically significant.

### Reporting summary

Further information on research design is available in the Nature Portfolio Reporting Summary linked to this article.

### Supplementary information

Supplementary Information

Description of Additional Supplementary Files

Supplementary Data

Reporting Summary

## Data Availability

The source data for graphs and the uncropped blots in the main text can be found in Supplementary Data and Supplementary Information (Supplementary Fig. 1). The data that support the findings of this study are available from the corresponding author upon reasonable request.
