# Supplementary material for: Inhibition of ACSL4 ameliorates tubular ferroptotic cell death and protects against fibrotic kidney disease
Source: Commun Biol. 2023 Sep 5;6:907. doi: 10.1038/s42003-023-05272-5 (PMC10480178; doi:10.1038/s42003-023-05272-5)
Supplement: Supplementary file 1 — Supplementary Information [file 42003_2023_5272_MOESM1_ESM.pdf]

Human		
Gene name		
COL1	Forward	GCGGACTTTGTTGCTGCTTGCAG
	Reverse	ATCTCCGGCTGGGCCCTTTCTT
α-SMA	Forward	ATCACCAACTGGGACGACAT
	Reverse	GGCAACACGAAGCTCATTG
FN	Forward	AATAGATGCAACGATCAGGACA
	Reverse	GCAGGTTTCCTCGATTATCCTT
TGF-β	Forward	CTGTACATTGACTTCCGCAAG
	Reverse	TGTCCAGGCTCCAAATGTAG
FGF2	Forward	CATCAAGCTACAACTTCAAGCA
	Reverse	CCGTAACACATTTAGAAGCCAG
CTGF	Forward	CAGCATGGACGTTCGTCTG
	Reverse	AACCACGGTTTGGTCCTTGG
PDGFB	Forward	GATCCGCTCCTTTGATGATCTC
	Reverse	GGTCATGTTCAGGTCCAACTC
GAPDH	Forward	ACCAAATCCGTTGACTCCGAC
	Reverse	CTCCTGTTCGACAGTCAGCC
Mus		
Gene name		
COL1	Forward	GGCAAAGATGGAGAAGCTGG
	Reverse	GGAAACCTCTCTCGCCTCTT
α-SMA	Forward	TGGCACCACACCTTCTACAA
	Reverse	CGGAGGCATAGAGGGACA
FN	Forward	ACAGTCCAGCAAGCAGCAAGC
	Reverse	TGGTGGTCACTCTGTAGCCTGTC
TGF-β	Forward	CCAGATCCTGTCCAAACTAAGG
	Reverse	CTCTTTAGCATAGTAGTCCGCT
FGF2	Forward	AGTTGTGTCTATCAAGGGAGTG
	Reverse	CATTGGAAGAAACAGTATGGCC
CTGF	Forward	AAAGCAGCTGCAAATACCAATG
	Reverse	AAATGTGTCTTCCAGTCGGTAG
PDGFB	Forward	GTCCAGGTGAGAAAGATTGAGA
	Reverse	GTCATGGGTGTGCTTAAACTTT
β-actin	Forward	GGTCAGAAGGACTCCTATGTGG
	Reverse	TGTCGTCCCAGTTGGTAACA

Supplementary Table 1: The primers sequence provided in 5′ → 3′ orientation

Supplementary information for the uncropped blots (Fig.1-Fig.9).

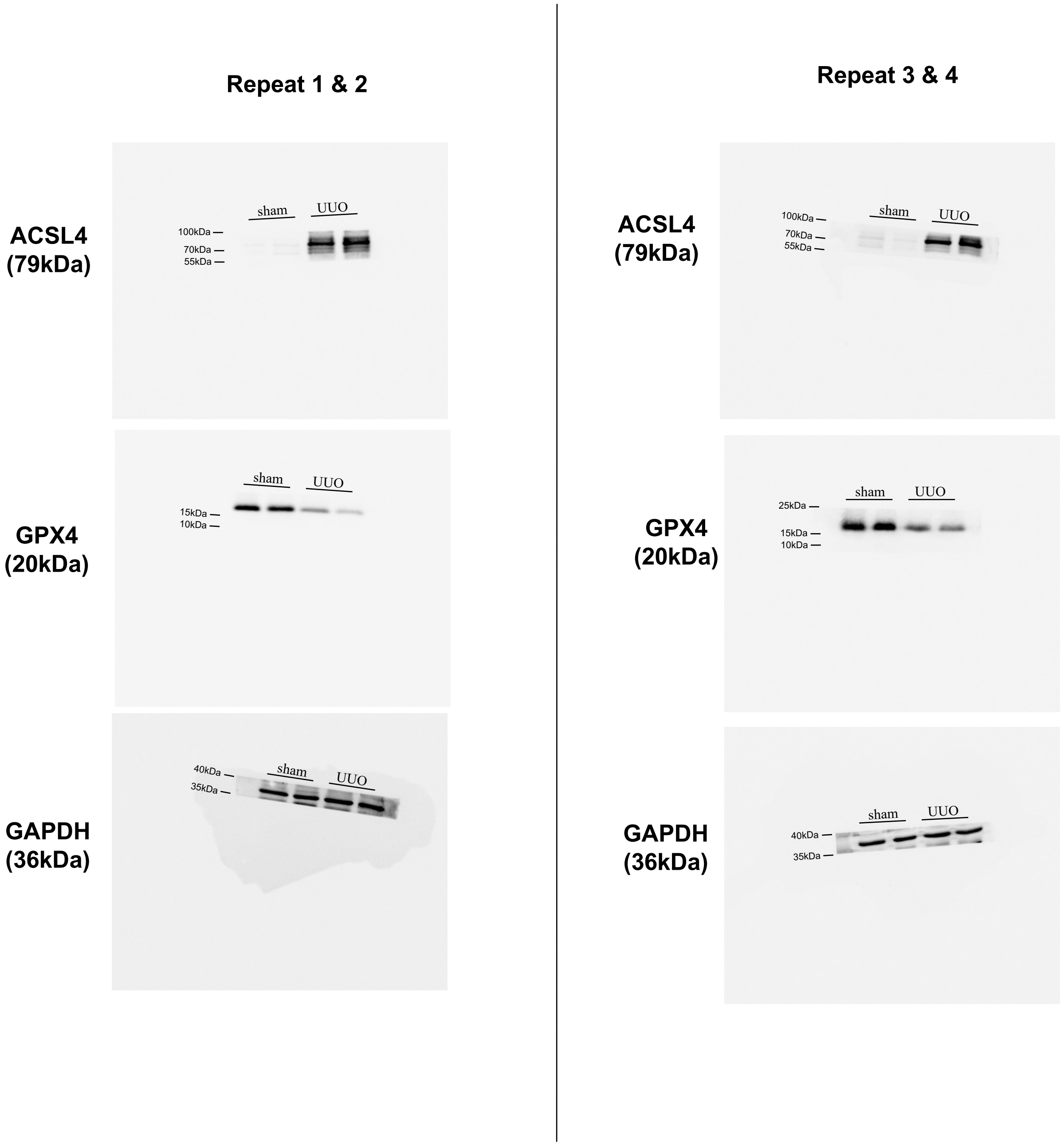

Supplementary Figure 1: The manuscript drawing for Fig.1c.

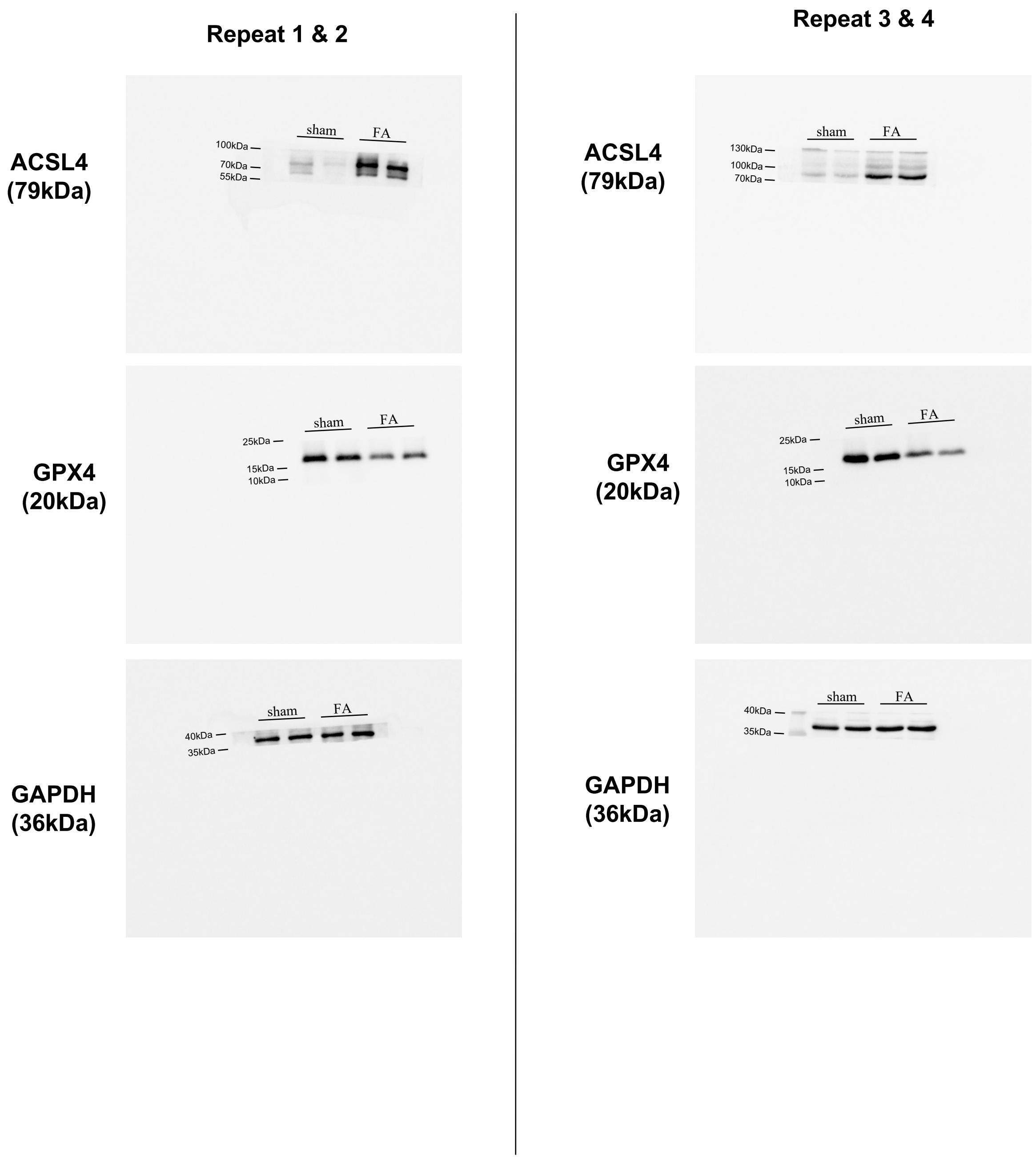

Supplementary Figure 1: The manuscript drawing for Fig.1d.

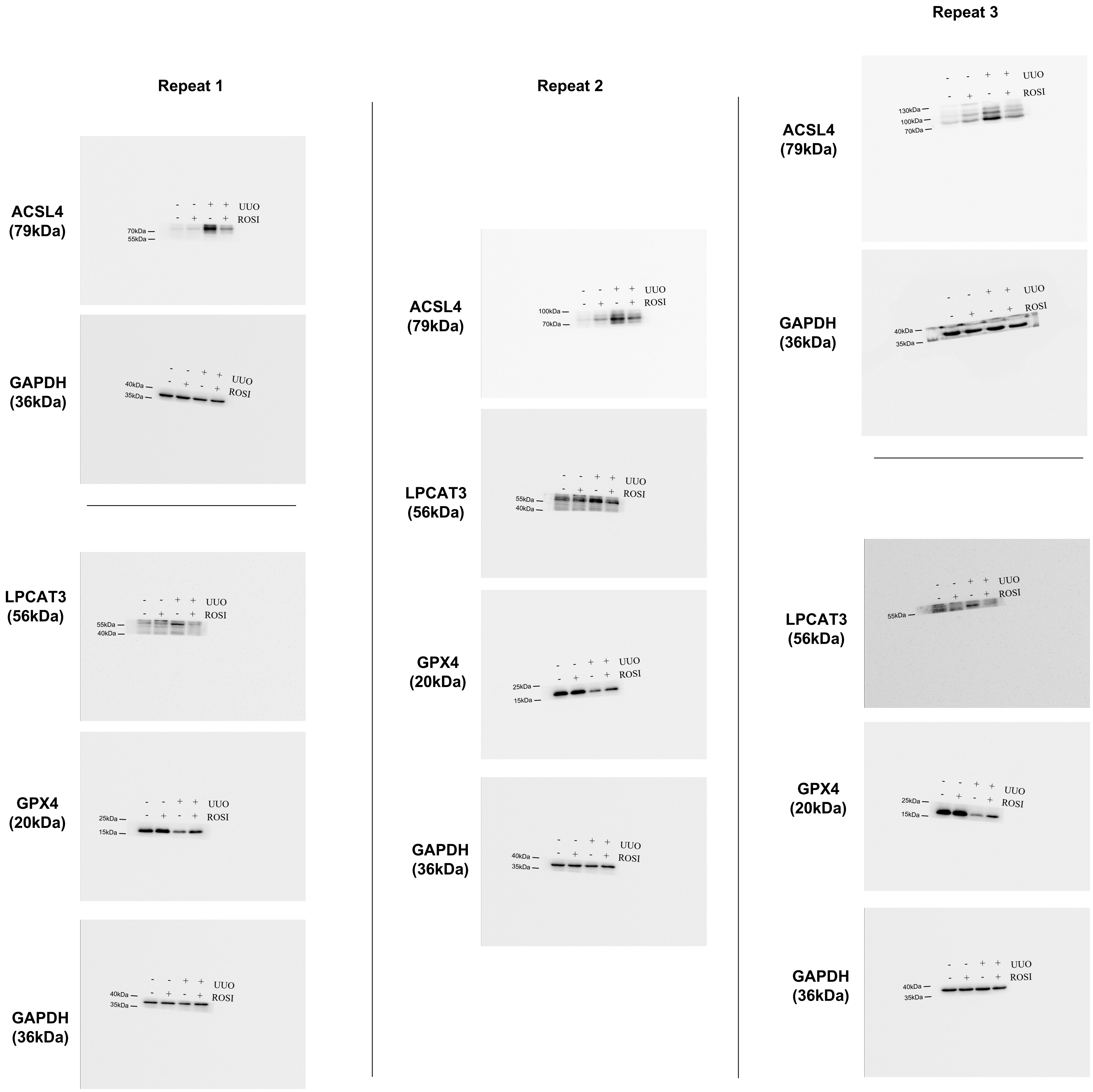

Supplementary Figure 1: The manuscript drawing for Fig.2b.

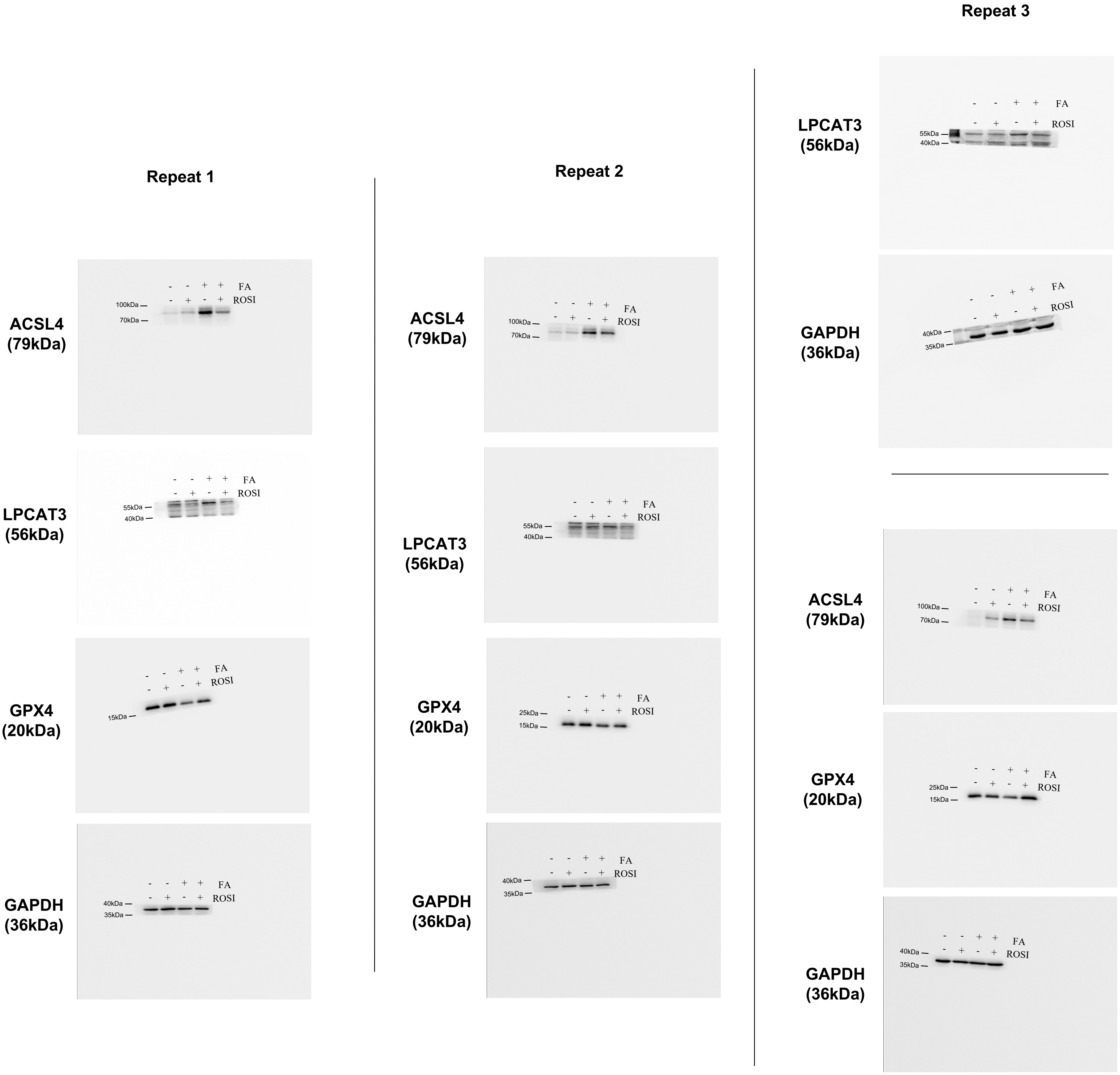

Supplementary Figure 1: The manuscript drawing for Fig.2c.

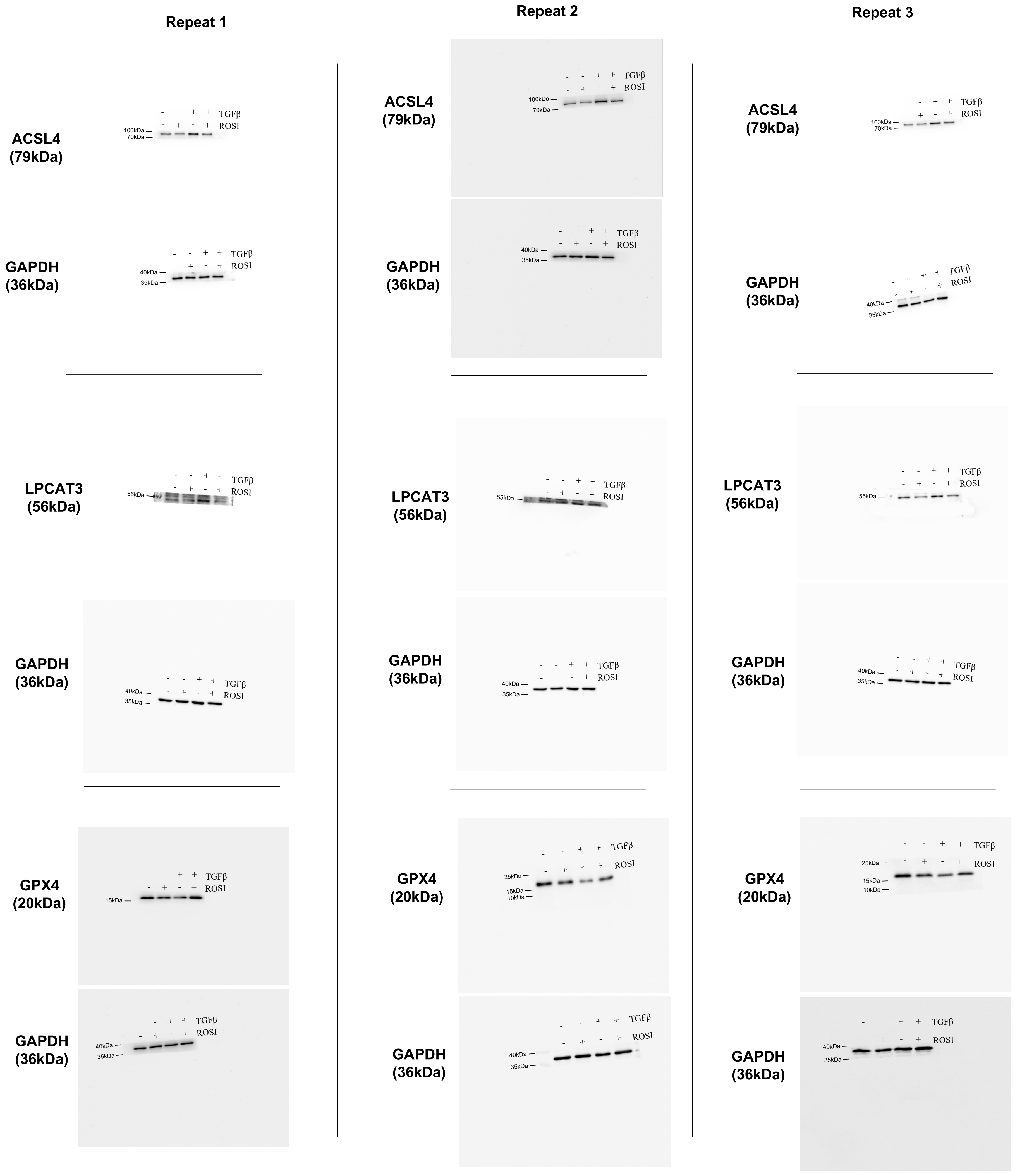

Supplementary Figure 1: The manuscript drawing for Fig.3b.

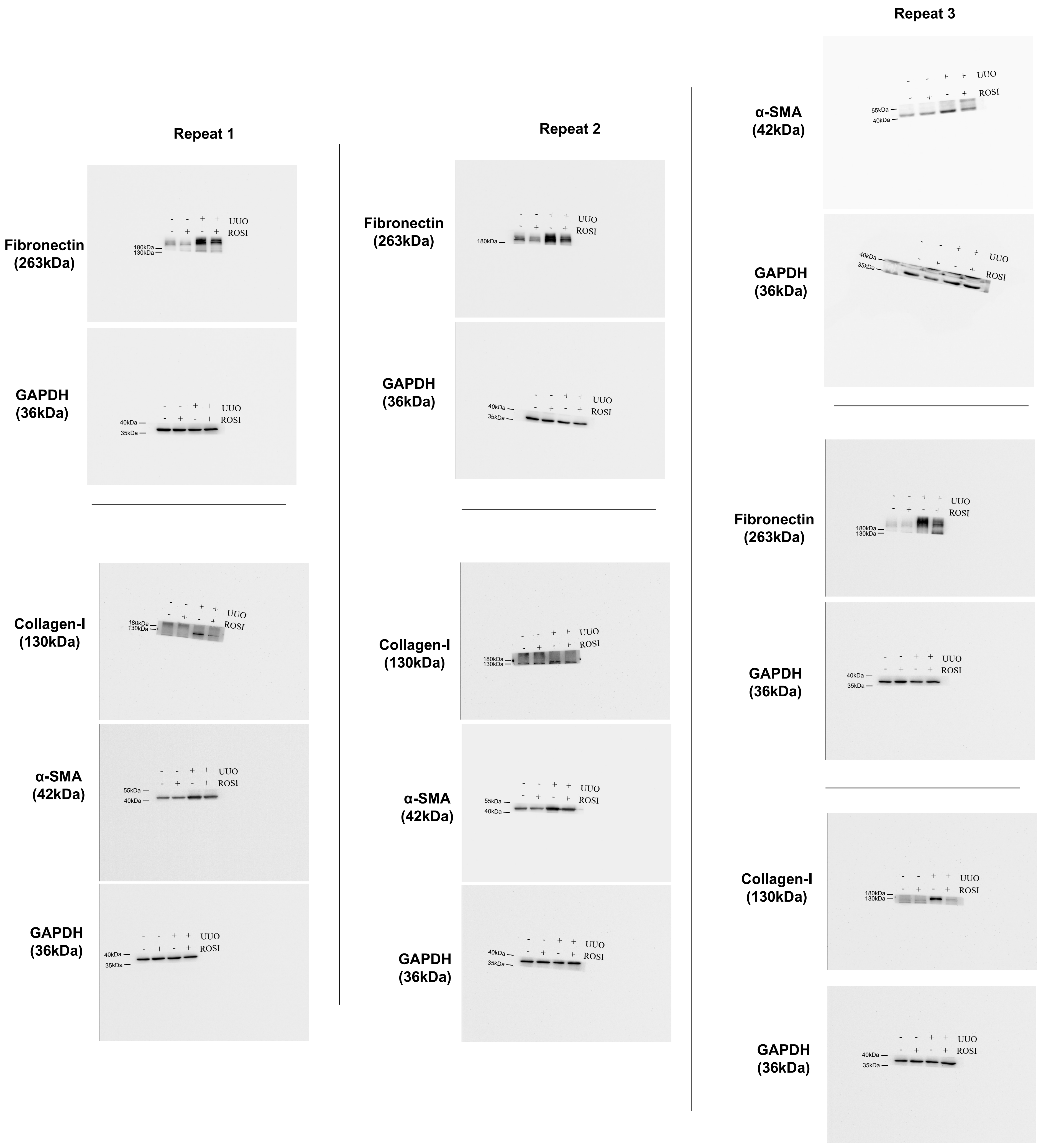

Supplementary Figure 1: The manuscript drawing for Fig.4b.

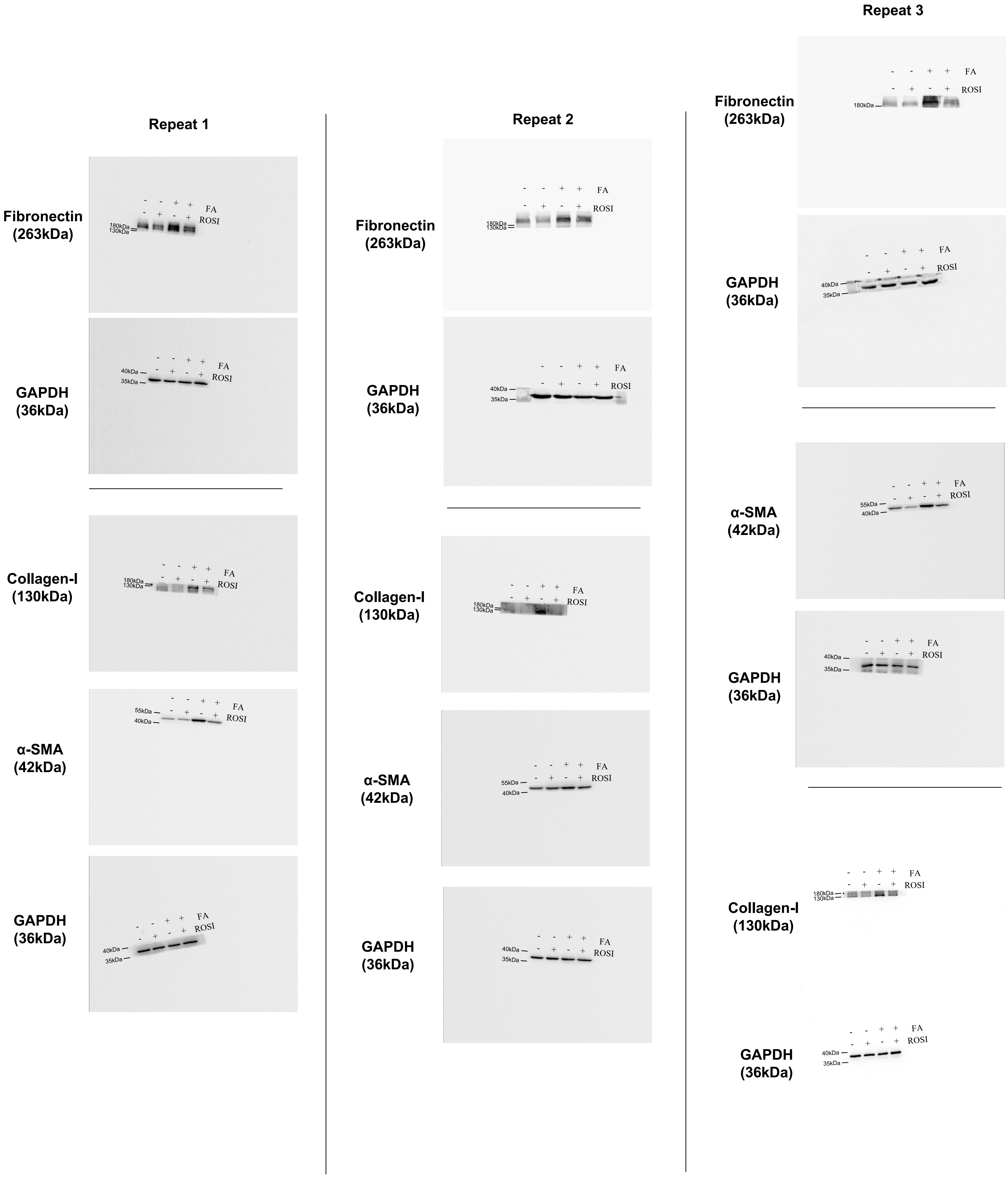

Supplementary Figure 1: The manuscript drawing for Fig.4c.

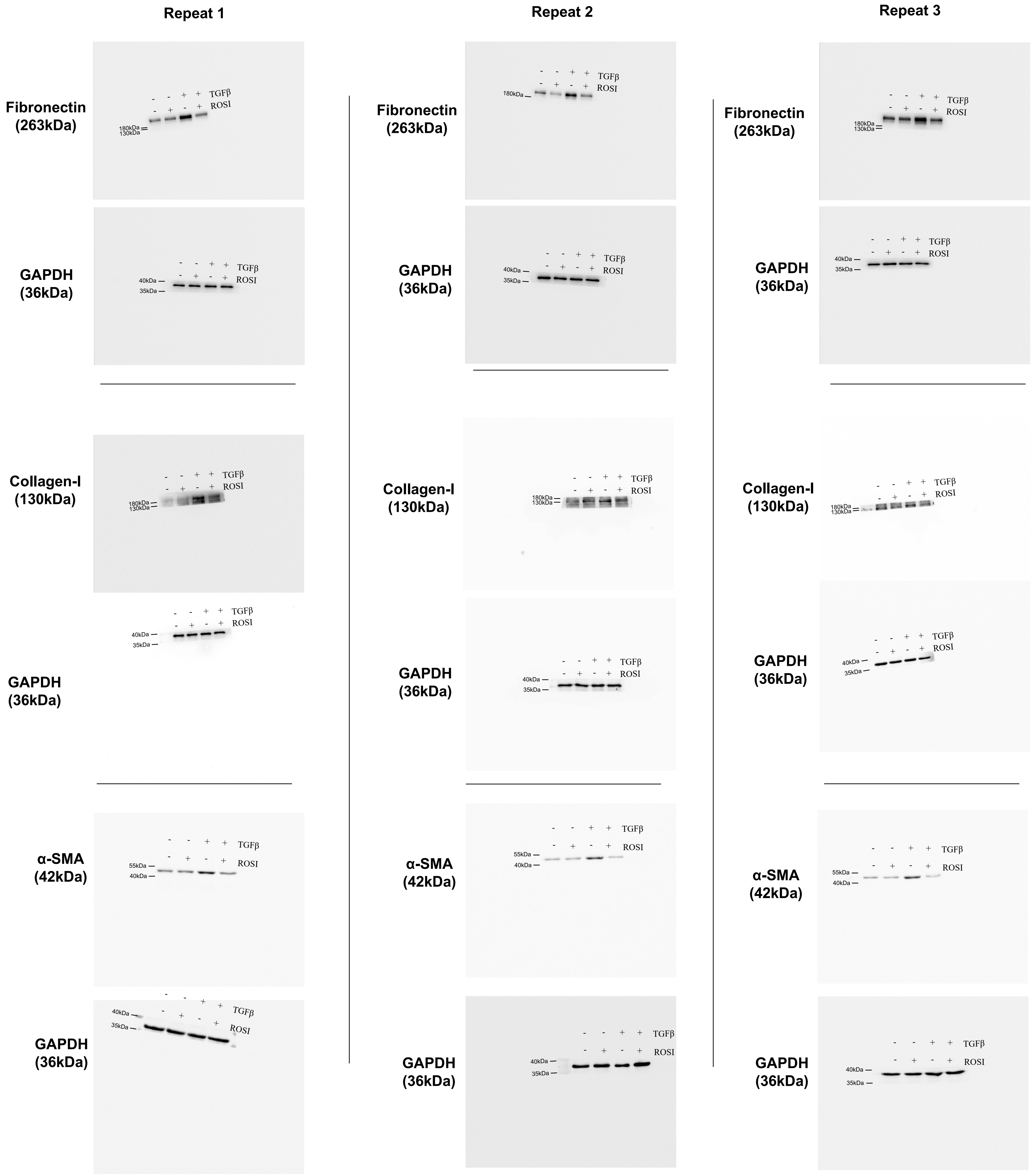

Supplementary Figure 1: The manuscript drawing for Fig.5b.

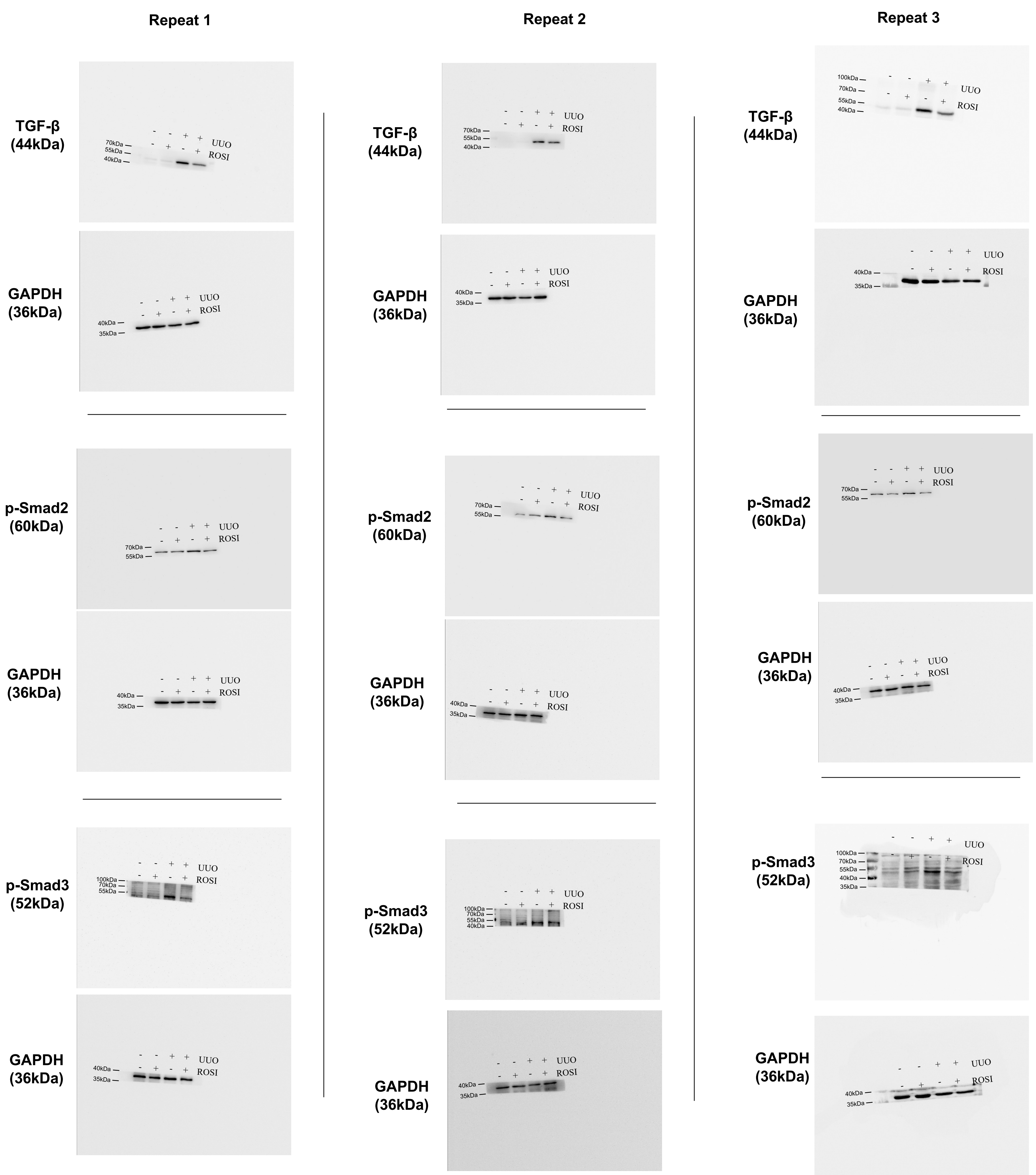

Supplementary Figure 1: The manuscript drawing for Fig.6a.

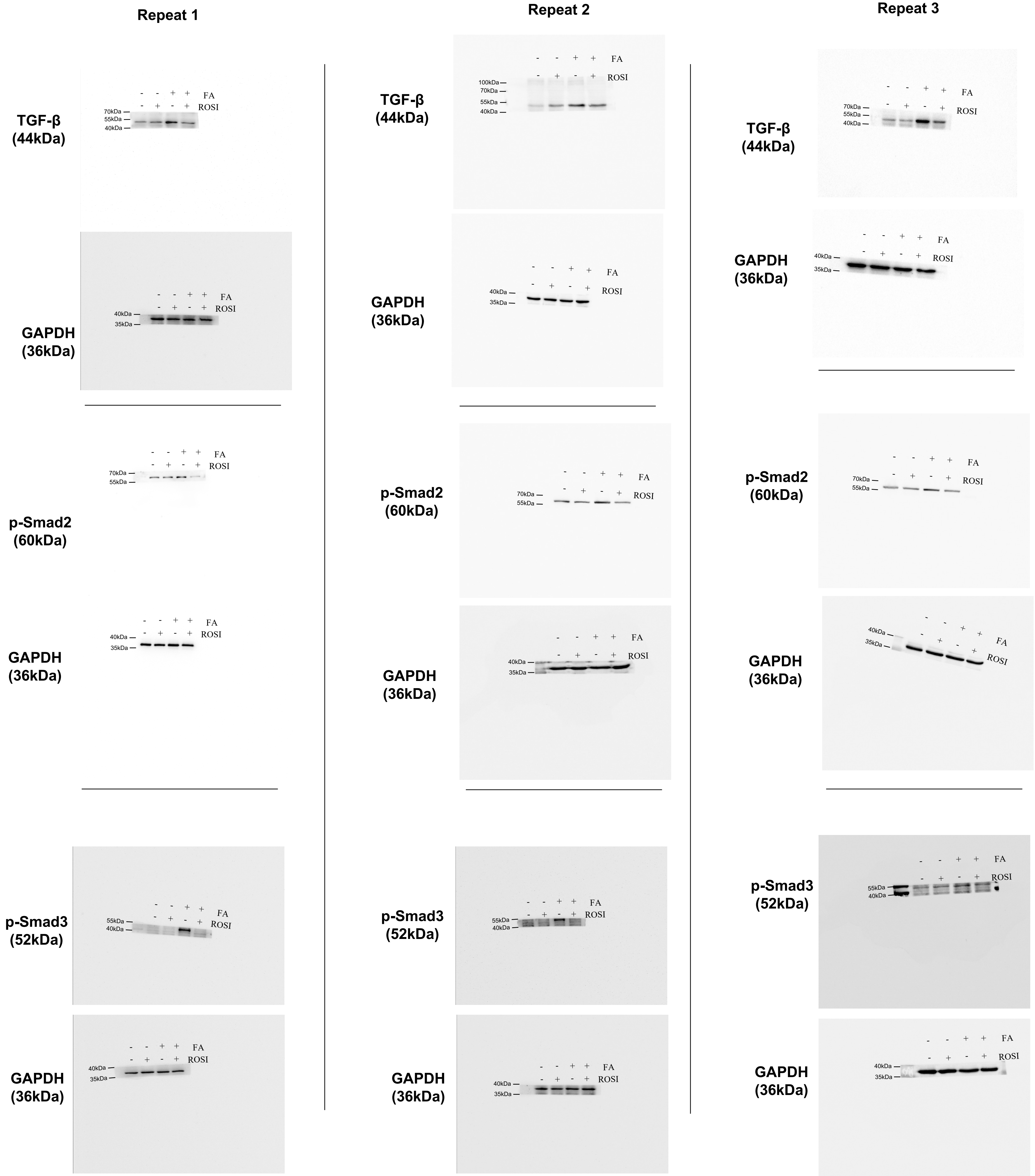

Supplementary Figure 1: The manuscript drawing for Fig.6b.

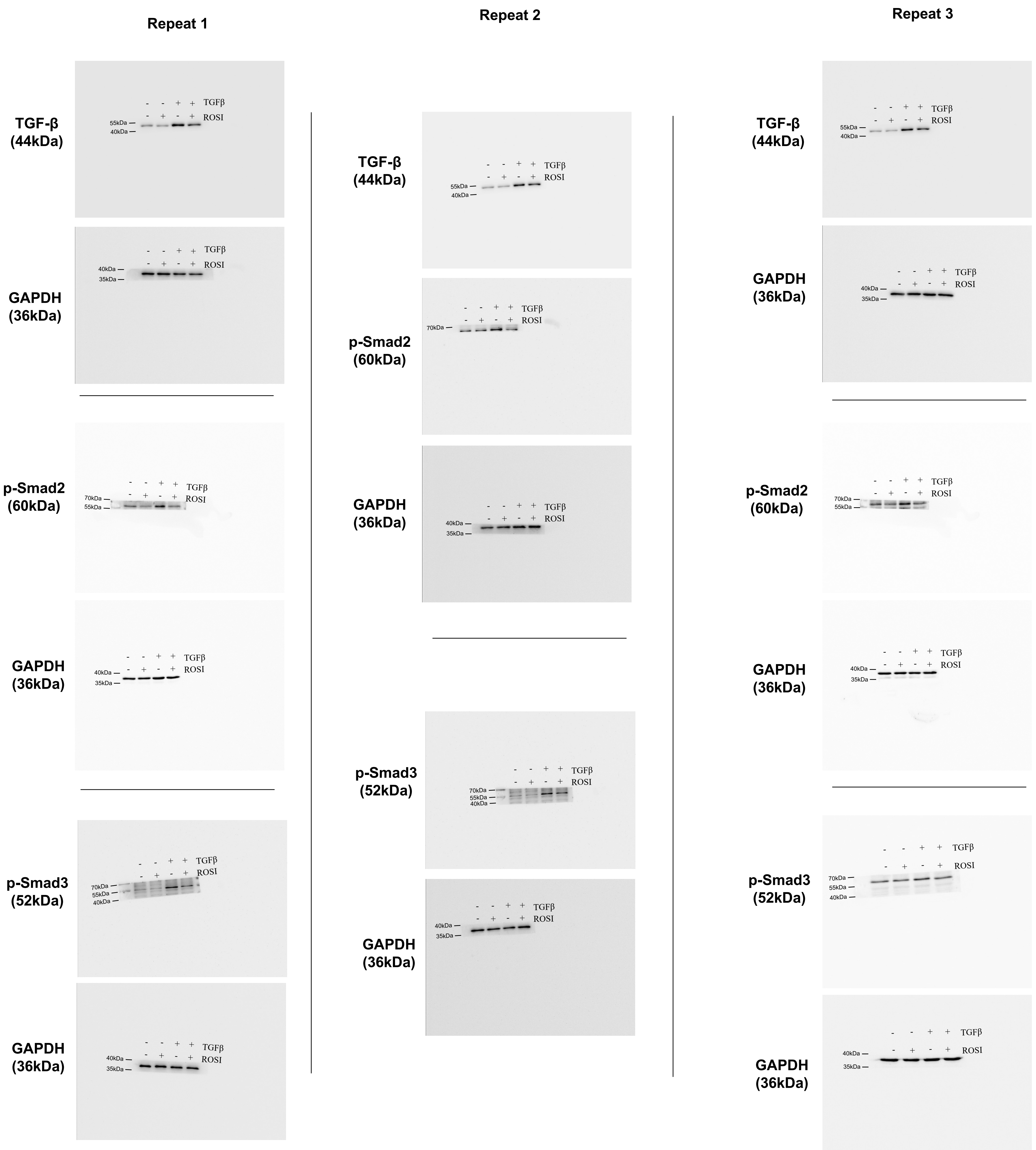

Supplementary Figure 1: The manuscript drawing for Fig.7a.

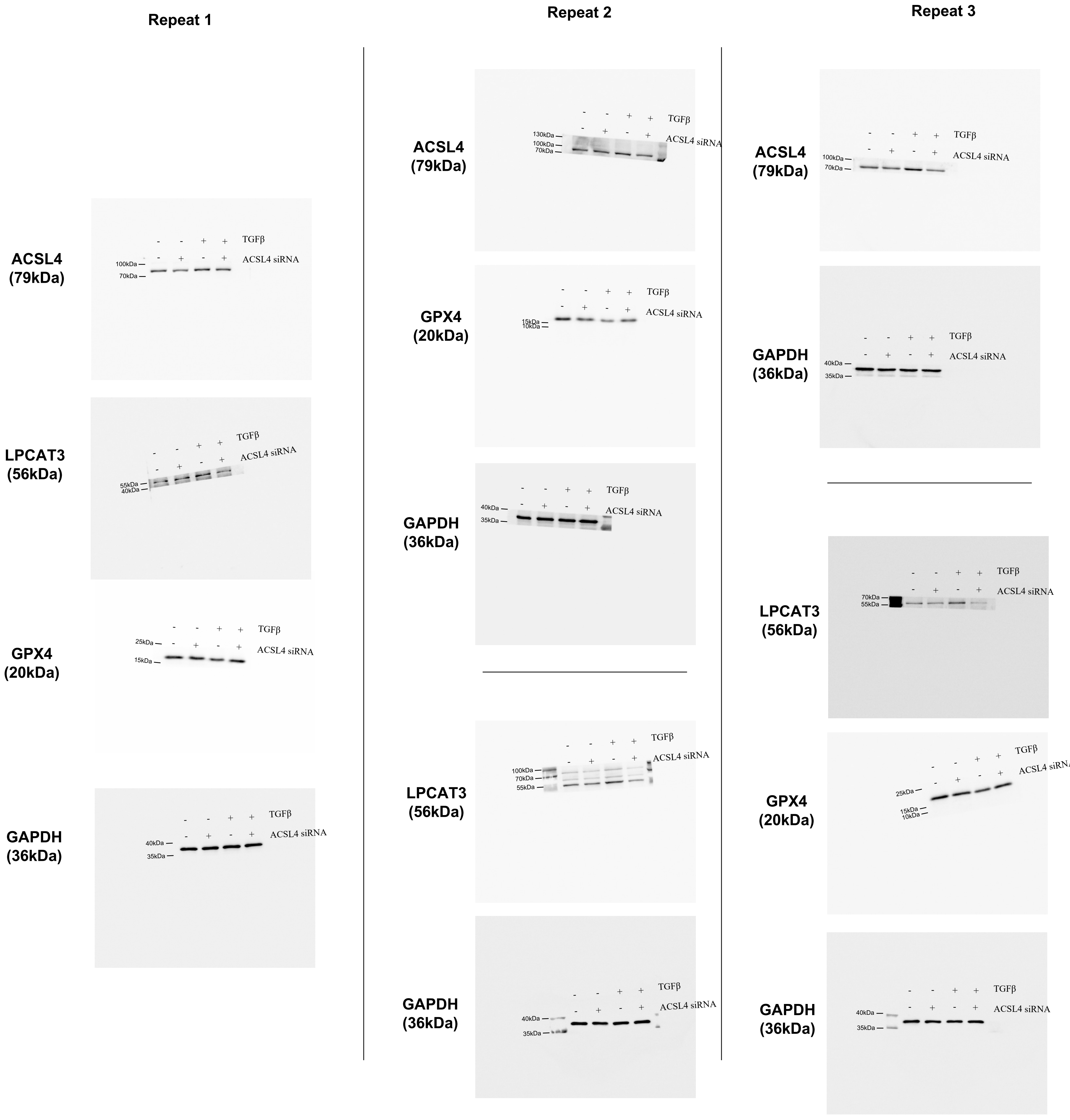

Supplementary Figure 1: The manuscript drawing for Fig.8b.

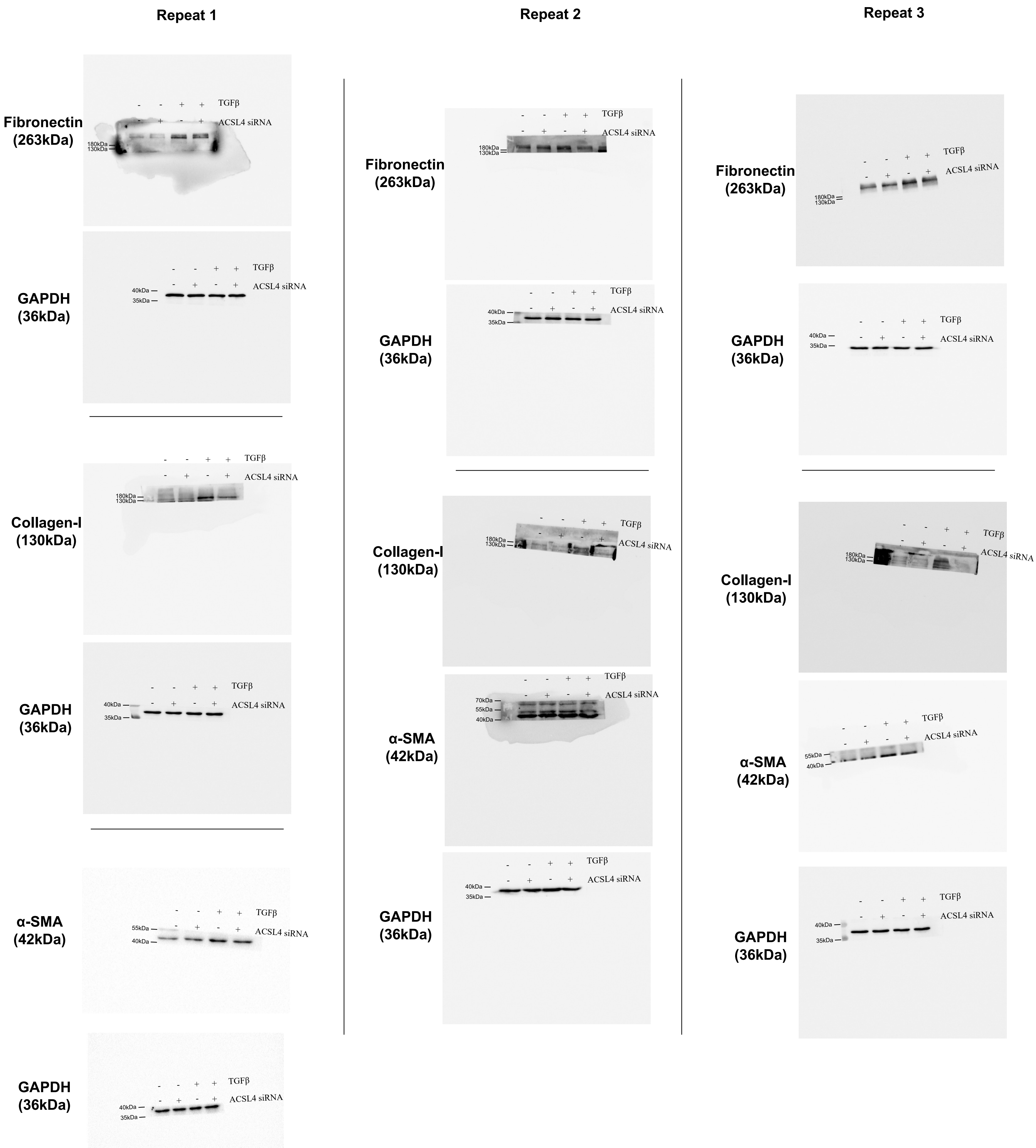

Supplementary Figure 1: The manuscript drawing for Fig.9b.
