# Supplementary material for: Inhibition of ACSL4 ameliorates tubular ferroptotic cell death and protects against fibrotic kidney disease
Source: Commun Biol. 2023 Sep 5;6:907. doi: 10.1038/s42003-023-05272-5 (PMC10480178; doi:10.1038/s42003-023-05272-5)
Supplement: Supplementary file 2 — Description of Additional Supplementary Files [file 42003_2023_5272_MOESM2_ESM.pdf]

Description of Additional Supplementary Files

File name: Supplementary Data 1
Description: The source data for graphs in the main text.
