# Supplementary material for: Inhibition of ACSL4 ameliorates tubular ferroptotic cell death and protects against fibrotic kidney disease
Source: Commun Biol. 2023 Sep 5;6:907. doi: 10.1038/s42003-023-05272-5 (PMC10480178; doi:10.1038/s42003-023-05272-5)
Supplement: Supplementary file 4 — Reporting Summary [file 42003_2023_5272_MOESM4_ESM.pdf]

nature portfolio

Corresponding author(s): Hongyu Gao, Qi Yan

Last updated by author(s): Aug 12, 2023

Reporting Summary

Nature Portfolio wishes to improve the reproducibility of the work that we publish. This form provides structure for consistency and transparency in reporting. For further information on Nature Portfolio policies, see our Editorial Policies and the Editorial Policy Checklist.

Statistics

For all statistical analyses, confirm that the following items are present in the figure legend, table legend, main text, or Methods section.

n/a	Confirmed	
☐	☒	The exact sample size (*n*) for each experimental group/condition, given as a discrete number and unit of measurement
☐	☒	A statement on whether measurements were taken from distinct samples or whether the same sample was measured repeatedly
☐	☒	The statistical test(s) used AND whether they are one- or two-sided<br>*Only common tests should be described solely by name; describe more complex techniques in the Methods section.*
☒	☐	A description of all covariates tested
☐	☒	A description of any assumptions or corrections, such as tests of normality and adjustment for multiple comparisons
☐	☒	A full description of the statistical parameters including central tendency (e.g. means) or other basic estimates (e.g. regression coefficient) AND variation (e.g. standard deviation) or associated estimates of uncertainty (e.g. confidence intervals)
☒	☐	For null hypothesis testing, the test statistic (e.g. *F*, *t*, *r*) with confidence intervals, effect sizes, degrees of freedom and *P* value noted<br>*Give P values as exact values whenever suitable.*
☒	☐	For Bayesian analysis, information on the choice of priors and Markov chain Monte Carlo settings
☒	☐	For hierarchical and complex designs, identification of the appropriate level for tests and full reporting of outcomes
☐	☒	Estimates of effect sizes (e.g. Cohen's *d*, Pearson's *r*), indicating how they were calculated

Our web collection on statistics for biologists contains articles on many of the points above.

Software and code

Policy information about availability of computer code

Data collection	no software was used
Data analysis	no software was used

For manuscripts utilizing custom algorithms or software that are central to the research but not yet described in published literature, software must be made available to editors and reviewers. We strongly encourage code deposition in a community repository (e.g. GitHub). See the Nature Portfolio guidelines for submitting code & software for further information.

Data

Policy information about availability of data

All manuscripts must include a data availability statement. This statement should provide the following information, where applicable:

- Accession codes, unique identifiers, or web links for publicly available datasets
- A description of any restrictions on data availability
- For clinical datasets or third party data, please ensure that the statement adheres to our policy

The data that support the findings of this study are available in the supplementary material of this article

Research involving human participants, their data, or biological material

Policy information about studies with human participants or human data. See also policy information about sex, gender (identity/presentation), and sexual orientation and race, ethnicity and racism.

Reporting on sex and gender	Human participants was not included in this study.
Reporting on race, ethnicity, or other socially relevant groupings	Human participants was not included in this study.
Population characteristics	Human participants was not included in this study.
Recruitment	Human participants was not included in this study.
Ethics oversight	Human participants was not included in this study.

Note that full information on the approval of the study protocol must also be provided in the manuscript.

Field-specific reporting

Please select the one below that is the best fit for your research. If you are not sure, read the appropriate sections before making your selection.

☒ Life sciences ☐ Behavioural & social sciences ☐ Ecological, evolutionary & environmental sciences

For a reference copy of the document with all sections, see nature.com/documents/nr-reporting-summary-flat.pdf

Life sciences study design

All studies must disclose on these points even when the disclosure is negative.

Sample size	The sample size was chosen to ensure that the statistical power were satisfied and that experimental effects were found while using as few animals as possible. The selection of sample sizes was based on previous publications and power analysis. The exact numbers of sample sizes were indicated in the figure legends.
Data exclusions	No data were excluded.
Replication	The different treatment groups in this study were repeated three times or more, and the specific reproducibility and related statistical analyses were presented in the figures. The study methods were described in detail in the "methods", and the important reagents were labeled with the company and product number.
Randomization	All mice were age and sex-matched and then randomized into the different groups based on the body weight.
Blinding	The researcher did not blind to the experimental groups. The analysis of the conclusions was based on the quantitative parameters and the statistical significance of objective data, which is independent of observer subjectivity and psychological expectations. Owing to the need to observe the viability and status of the mice during modeling and drug administration. Thus, the experimental observations were independent of blinding.

Reporting for specific materials, systems and methods

We require information from authors about some types of materials, experimental systems and methods used in many studies. Here, indicate whether each material, system or method listed is relevant to your study. If you are not sure if a list item applies to your research, read the appropriate section before selecting a response.

Materials & experimental systems

n/a	Involved in the study	
☐	☒	Antibodies
☐	☒	Eukaryotic cell lines
☒	☐	Palaeontology and archaeology
☐	☒	Animals and other organisms
☒	☐	Clinical data
☒	☐	Dual use research of concern
☒	☐	Plants

Methods

n/a	Involved in the study	
☒	☐	ChIP-seq
☒	☐	Flow cytometry
☒	☐	MRI-based neuroimaging

Antibodies

Antibodies used	The source of antibodies is reported in "Western Blot and Antibody". Rabbit anti GPX4 (Abcam, ab125066, 1:3000 dilution for WB, 1:100 dilution for IHC, Britain), Mouse anti ACSL4 (Santa Cruz, SC393906, 1:1000 dilution for WB, USA), Rabbit anti LPCAT3(Abcam, ab232958, 1:500 dilution for WB, Britain), Rabbit anti Fibronectin (Abcam, ab45688, 1:5000 dilution for WB, 1:250 dilution for IF, Britain), Rabbit anti α-SMA (Abcam, ab124964,1:2000 dilution for WB, 1:250 dilution for IF, Britain), Rabbit anti Collagen-Ⅰ (Proteintech, 14695-1-AP,1:1000 dilution for WB, USA), Rabbit anti TGF-β1 (Abcam, ab215715, 1:1000 dilution for WB, Britain), Rabbit anti p-Smad2 (CST, 3104 S,1:1000 dilution for WB, USA), Rabbit anti p-Smad3(CST, 9520T S,1:1000 dilution for WB, USA), Rabbit anti 4-HNE (Abcam, ab46545, 1:100 dilution for IF, Britain) and Rabbit anti GAPDH (Promoter, 1:25000 for WB, China).
Validation	The primary validation antibodies not used in this study.

Eukaryotic cell lines

Policy information about cell lines and Sex and Gender in Research

Cell line source(s)	HK-2 (human kidney tubular cell) was obtained from China Centre for Type Culture Collection (CCTCC, China), and maintained in DMEM-F12 (Biological Industries, USA) medium containing 10% fetal bovine serum (FBS) and 1% penicillin-streptomycin. Cells were incubated in a humidified atmosphere of 95% air and 5% CO_2 at 37°C.
Authentication	HK-2 cells were taken for DNA extraction. Then, the DNA was amplified and identified using the STR method and analyzed using a PCR instrument. The results were compared with the ATCC, DSMZ and Cellosaurus databases. The DNA amplification plot was clear, and no cross-infection of other human cells was found. The DNA typing of this cell line found a 100% match to its cell typing in the database, with the cell line name HK-2.
Mycoplasma contamination	Cell lines were not tested for mycoplasma contamination. All cell lines tested negative for mycoplasma contamination.
Commonly misidentified lines (See ICLAC register)	Commonly misidentified cell lines was not used in this study,

Animals and other research organisms

Policy information about studies involving animals; ARRIVE guidelines recommended for reporting animal research, and Sex and Gender in Research

Laboratory animals	Male 8–12-week C57BL/6J mice were fed in a specific pathogen-free (SPF) environment in the laboratory animal center.
Wild animals	This study did not involve in wild animals.
Reporting on sex	In this study, a single sex of male mice was used for the reason that the hormone levels in male mice are more stable, which ensures the objectivity of the experiment and reduces the chance error in animal experiments.
Field-collected samples	Animals were given free access to normal diet and acclimatized in a ventilated temperature-controlled room (24 °C), with a regular 12 h light/dark cycle.For unilateral ureteral obstruction (UUO) model, mice underwent left UUO surgery, the left mid-ureter of mice was exposed via a lateral incision and obstructed it twice with 4–0 silk sutures.For folic acid administration model, mice were intraperitoneally injected with a single dose of folic acid (250 mg/kg body weight dissolved in 300 mM $NaHCO_3$). The mice were intraperitoneally treated with ROSI (0.5 mg/kg/day) at 1h prior to handlings of UUO and FA, and continuously injected daily for UUO and FA duration. After 14 days for UUO model and 28 days for FA model, kidney tissues were collected for the further experiments.
Ethics oversight	This paper does not describe studies involving human participants, human data or human tissue. All animal interventions were approved by the Institutional Animal Care and Use Committee(No. S 2670).

Note that full information on the approval of the study protocol must also be provided in the manuscript.
